# Crystallographic and Computational Analysis of Oxyma B Cocrystals with Nitrogen-Containing Coformers: The Relevant Role of *n* → π* Interactions in Their Diverse Supramolecular Architectures

**DOI:** 10.1021/acs.cgd.5c00884

**Published:** 2025-09-25

**Authors:** Mahdi Jemai, Rafael Barbas, Miquel Barceló-Oliver, Houda Marouani, Fernando Albericio, Antonio Frontera, Rafel Prohens

**Affiliations:** † Laboratory of Organic Chemistry, Faculty of Pharmacy and Food Sciences, University of Barcelona, Avda. Joan XXIII Barcelona 08028, Spain; ‡ Laboratory of Material Chemistry, LR13ES08, Faculty of Sciences of Bizerte, 61796 University of Carthage, Bizerte 7021, Tunisia; § Unitat de Polimorfisme i Calorimetria, Centres Científics i Tecnològics, 16724 Universitat de Barcelona, Baldiri Reixac 10, Barcelona 08028, Spain; ∥ Department of Chemistry, University of Balearic Islands, Palma 07122, Spain; ⊥ Department of Organic Chemistry, University of Barcelona, Martí i Franqués 1-11, Barcelona 08028, Spain; # School of Chemistry and Physics, 56394 University of KwaZulu-Natal, Durban 4001, South Africa

## Abstract

Three new cocrystals of Oxyma-B, an important racemization suppressor for peptide synthesis, with 6-methylquinoline (I), 2,3,5,6-tetramethylpyrazine (II), and 1,10-phenanthroline (III) were synthesized and their single crystal structures analyzed. They show a rich network of noncovalent interactions, including classical and nonclassical hydrogen bonds (CH···O, OH···N, CH···N), CH···π, π-stacking, and, notably, lone pair···π (*n* → π*) interactions. Distinctive supramolecular synthons were identified, including the R_2_^2^(7) motif found in both 6-methylquinoline/Oxyma-B and 2,3,5,6-tetramethylpyrazine/Oxyma-B cocrystals. In 1,10-phenanthroline/Oxyma-B, larger ring motifs such as R_4_^4^(20) and R_5_^5^(24) were observed, further supported by additional synthons of types *R*_1_^2^(5) and *R*_1_^2^(6). Hirshfeld surface analysis and density functional theory (DFT) calculations, including MEP surface, QTAIM, and NCIplot analyses, were carried out to quantify the intermolecular contributions and rationalize the experimental findings with a focus on the cooperative role of hydrogen bonding, π-stacking, and lone pair···π (*n* → π*) interactions in stabilizing and shaping the architectures of these new multicomponent crystalline materials.

## Graphical abstract

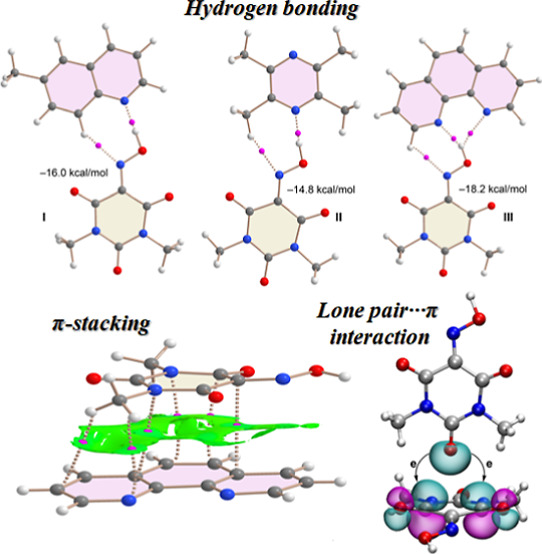

## 1 Introduction

Oximes (RR’C = NOH) represent a multifunctional class of organic compounds with a wide range of commercial applications. They are widely used in pharmaceuticals, natural products, heterocycles, and polymer industries.^1^ Within oximes family, Oxyma-Pure and its derivatives constitute an important subclass, particularly noted for their use as coupling additives in peptide synthesis.^2^ Oxyma-Pure offers excellent reactivity as an additive that efficiently suppresses racemization during amide bond formation.^3^ Recently, efforts to enhance the properties of Oxyma led to its development and therefore the synthesis of its cyclic version, known as Oxyma-B.^4^ Oxyma-B has demonstrated even greater efficiency in inhibiting racemization compared to the parent Oxyma compound.^4^

In the field of solid-state Chemistry, the study of the intermolecular interactions that govern the packing and the arrangement of molecules in the crystal lattice is essential to understand the properties of the different solid forms of a compound. The study of NCIs such as hydrogen bonding, π-stacking, and lone pair···π interactions plays a crucial role in crystal engineering, influencing the design, stability, and properties of crystalline materials.^5,6^ In particular, lone pair···π contacts have emerged as important contributors to supramolecular assembly and stabilization of crystal packing, showing that these interactions can be exploited in the rational design of functional cocrystals.^5,7^ This interaction occurs when a compound with unsaturated surfaces which are locally electron deficient show positive electrostatic potential on a region known as π-hole with the potential to interact with electron rich atoms. It is important to mention that this type of intermolecular interactions is not exclusive of aromatic compounds and they have been observed in nonaromatic donors containing an electron deficient π-system.^8^ The work by Molčanov et al. systematically studied π interactions between nonaromatic rings, revealing that the strongest interactions are between rings with little or no electron delocalization (such as quinones).^9^

A particular case of lone pair···π interactions involving carbonyl groups is the so-called *n* → π* interaction. This attractive force was originally studied by Bürgi and Dunitz during the analysis of addition reactions of a nucleophile to a carbonyl group^10^ and have been extensively studied as an intramolecular stabilizing force in proteins and peptides, dictating their tridimensional structure. It can be defined as the donation of electron density from a lone-pair orbital of a nucleophile to the empty π* orbital of a nearby carbonyl group.^11^

In spite of being an interaction well studied in intramolecular processes of protein folding,^12^ its occurrence in supramolecular systems, like cocrystals, is less known. Recently, barbituric acid derivatives, a family of pharmacologic compounds with sedatives, anticonvulsants, hypnotics, and antihypertensives properties and containing three carbonyl groups in a cyclic system has been used by Wang and co-workers as a model for the study of the *n* → π* interaction.^13,14^

In this context, Oxyma-B, a nonaromatic pyrimidinetrione compound derived from barbituric acid with an electron deficient region on the ring prone to act as an acceptor in lone pair···π interactions, has been the subject of previous research by some of us due to its ability to engage in diverse noncovalent interactions (NCIs), revealing that Oxyma-B forms rich hydrogen bonding networks and strong lone pair···π (O···π) interactions.^15^ The study emphasized the essential role of short O···π contacts (distances <3 Å) in stabilizing the crystal lattice of Oxyma-B. However, despite its potential in supramolecular chemistry, systematic exploration of Oxyma-B’s behavior in multicomponent crystals remains very limited, offering an exciting opportunity for further supramolecular studies. The literature shows that, to date, the only example where Oxyma-B participates in crystalline supramolecular assembly is the research work of Orlandin et al. focused on peptide synthesis protocols and its ability to enable amide bond formation in impressive yields, eliminate loss of chirality as well as block racemization.^16^

To further explore the supramolecular capabilities of Oxyma-B, we selected three organic coformers 6-methylquinoline, 2,3,5,6-tetramethylpyrazine, and 1,10-phenanthroline based on their structural features and potential for diverse noncovalent interactions, particularly as H-bond acceptors and as donors in lone pair···π and *n* → π* interactions. In particular, 6-methylquinoline offers an aromatic system capable to establish π-stacking and CH···π interactions, while its nitrogen atom can participate in hydrogen bonding interactions. 2,3,5,6-Tetramethylpyrazine provides a rigid, planar π-system with multiple methyl groups and two nitrogen atoms positioned for strong hydrogen bonding, as well as for 1,10-phenanthroline, in addition to their ability to be bound via strong hydrogen bond interactions, the three planar aromatic rings present a fertile system for different types of π interactions. The common but differentiated features of the three coformers were expected to generate rich supramolecular architectures when combined with the versatile hydrogen bonding and lone pair···π donor sites of Oxyma-B and are the subject of the work presented herein.

The new cocrystals of Oxyma-B were analyzed through a joint experimental/computational approach by combining single-crystal X-ray diffraction (SCXRD) and computational tools, including molecular electrostatic potential (MEP) surface analyses, quantum theory of atoms in molecules (QTAIM), and noncovalent interaction (NCI) plot analyses, in order to rationalize the crystal packing and to highlight the crucial contributions of hydrogen bonding, π-stacking, and particularly lone pair···π interactions in stabilizing the supramolecular architectures.

## 2 Experimental Section

### 2.1 Synthesis and Crystallization

The cocrystals were synthesized via the slow evaporation method. Equimolar amounts of Oxyma-B (50 mg) and the respective coformer; 6-methylquinoline, 1,10-phenanthroline and 2,3,5,6-tetramethylpyrazine were dissolved in a mixed solvent system consisting of ethanol, acetone, and tetrahydrofuran (THF). The solutions were stirred for 30 min at 40 °C. The homogeneous solutions obtained were allowed to evaporate slowly at room temperature. After 3–4 days, single crystals suitable for SCXRD analysis were obtained for each cocrystal and collected by filtration, washed quickly with cold ethanol, and dried under ambient conditions.

### 2.2 Crystal Structure Determination

The single-crystal X-ray diffraction data for the three newly synthesized cocrystals were collected using a Bruker D8 VENTURE diffractometer equipped with an Incoatec IμS DIAMOND microfocus Cu Kα radiation source (λ = 1.54178 Å) and Incoatec Helios MX multilayer optics. Data reduction and cell refinement were performed with the Bruker APEX5 software package.^17^ Absorption corrections were applied using the SADABS-2016/2 multiscan method.^17^ The crystal structures were solved by intrinsic phasing with SHELXT-2018/2 and refined using full-matrix least-squares refinement on F^2^ with SHELXL-2019/3, all within the Olex2–1.5 suite.^18,19^ All non-hydrogen atoms were refined anisotropically. Hydrogen atoms bonded to oxygen were located from the difference Fourier map and refined freely, while those bonded to carbon were placed in geometrically calculated positions and refined using a riding model with U_iso_(H) = 1.2U_eq_(C).

For compound II, the oxyma B molecule shows rotational disorder, being found in two nonequivalent positions (ca. 70 and 30% occupancy). In that case, some restraints (DFIX and DANG) were used to fix the hydroxy proton once located in the Fourier difference map.

Each structure was also checked for possible higher symmetry using the PLATON software.^20^ The crystallographic data of the three cocrystals are summarized in Table 1.

**Table 1:** Crystallographic Data of Cocrystals I, II and III

compound	I	II	III
empirical formula	C_6_H_6_N_3_O_4_·C_10_H_10_N	C_6_H_7_N_3_O_4_·0.5(C_8_H_12_N_2_)	C_6_H_7_N_3_O_4_·C_12_H_8_N_2_
formula weight	328.33	253.24	365.35
temperature (K)	299	100	100
crystal system	monoclinic	monoclinic	orthorhombic
space group	P2_1_ */n*	P2_1_ */n*	*P*2_1_2_1_2_1_
*a* (Å)	11.9251 (11)	9.8437 (7)	14.7756 (14)
*b* (Å)	8.4682 (8)	6.1775 (4)	15.1618 (14)
*c* (Å)	16.0576 (15)	18.7477 (13)	7.3586 (7)
α (°)	90	90	90
β (°)	108.274 (4)	101.465 (3)	90
γ (°)	90	90	90
*V* (Å^3^)	1539.8 (3)	1117.29 (13)	1648.5 (3)
*Z*	4	4	4
density (calc. Mg/m^3^)	1.416	1.506	1.472
final R indices [I > 2σ(I)]	R1 = 0.048 W R2 = 0.133	R1 = 0.070 W R2 = 0.177	R1 = 0.056 W R2 = 0.150
CCDC	2464146	2464147	2464172

### 2.3 Theoretical Methods

Density functional theory (DFT) calculations of the supramolecular assemblies were carried out using the PBE0 functional with Grimme’s D3 dispersion correction and the def2-TZVP basis set, as implemented in the Gaussian 16 software package.^21–24^ Binding energies were calculated as the difference between the total energy of the complex and the sum of the energies of the isolated monomers, with counterpoise corrections applied to account for basis set superposition error (BSSE).^25^ Molecular electrostatic potential (MEP) surfaces were computed on the 0.001 au electron density isosurface, which closely approximates the van der Waals surface. The Oxyma-B···N-donor cocrystallized molecular pairs were fully optimized without symmetry constraints. All optimized structures were confirmed as true minima on the potential energy surface via vibrational frequency analysis.

To analyze cooperativity effects within the assemblies, the quantum theory of atoms in molecules (QTAIM)^26^ and noncovalent interaction plot (NCIPlot)^27^ methods were employed at the same level of theory and using the X-ray coordinates. QTAIM analyses were performed using the AIMAll software package.^28^

The NCIPlot method, which visualizes noncovalent interactions in real space, utilizes the reduced density gradient (RDG)^29^ in combination with the sign of the second eigenvalue (λ_2_) of the electron density Hessian to distinguish between attractive and repulsive interactions. In this study, the following NCIPlot parameters were used: RDG = 0.5, electron density cutoff = 0.04 au, and a color scale ranging from −0.04 au ≤ sign­(λ_2_)­ρ ≤ 0.04 au In the resulting plots, strongly attractive interactions are shown in blue, strongly repulsive interactions in red, weak attractive interactions in green, and weak repulsive interactions in yellow.

Natural bond orbital (NBO) analysis was carried out using the NBO 7.0 program^30^ at the PBE0-D3/def2-TZVP level of theory to examine donor–acceptor interactions. In particular, second-order perturbation theory was employed to quantify the stabilization energies associated with lone pair (*n*) to antibonding (π*) orbital charge transfer interactions.

## 3 Results and Discussion

Each of the three Oxyma-B cocrystals examined in this study exhibit distinct crystallographic characteristics. While their packing motifs are different, the common feature is the complex network of weak interactions characterized by the presence of directional forces such as π-stacking, lone pair···π interactions, and hydrogen bonds. The following analysis provides deep structural insight into how Oxyme-B adapts to diverse chemical environments, forming a rich and diverse supramolecular landscape.

### 3.1 Structural Description and Supramolecular Details

#### 3.1.1 6-Methylquinoline/Oxyma-B Cocrystal (I)

6-methylquinoline/Oxyma-B cocrystal (I) crystallizes in the Monoclinic crystal system with the space group *P*2_1_/*n* and with one molecule of each component in the asymmetric unit, Figure S1. The Oxyma-B molecule exhibits positional disorder of the hydrogen atoms bonded to C3 and C5, with refined occupancies of 0.62/0.38 and 0.57/0.43, respectively. For a clearer understanding of the supramolecular organization, the analysis was performed considering only the major disorder component of the Oxyma-B molecule.

The crystal packing of I is displayed along the ( a⃗,c⃗) plane in Figure S2a. The structure reveals the formation of extended supramolecular ribbon consolidated by hydrogen bonds. Figure S2b provides a close-up view highlighting the key H-bonds interactions in the cocrystal. A strong OH···N hydrogen bond is observed between the oxyme group and the nitrogen atom of 6-methylquinoline (O1–H1···N1A), serving as the strongest and expected interaction in the assembly. In addition, weak CH···O (C11A-H11C···O4) and (C8A-H8A···N1) H-bonds in turn strengthens the attachment, giving rise to an R_2_^2^(7) supramolecular synthon,^31,32^ enhancing the overall consolidation and directionality of the supramolecular architecture. The geometric parameters of the H-bonds are given in Table 2.

**Table 2:** Hydrogen-Bonds Geometry in Cocrystal I (Å, °)^a^

D—H···A	D—H (Å)	H···A (Å)	D···A (Å)	D—H···A (°)
O1—H1···N1A	1.32 (3)	1.36 (3)	2.648 (2)	161 (3)
C8A—H8A···N1	0.93	2.94	3.695 (3)	140
C11A—H11C···O4^i^	0.96	2.57	3.313 (3)	135
C5—H5D···O2^ii^	0.96	3.01	3.953 (3)	168
C3—H3D···O4^ii^	0.96	2.75	3.654 (3)	158

a Symmetry codes: (i) −*x* + 1/2, *y* + 1/2, −*z* + 1/2; (ii) −*x* + 1/2, *y* – 1/2, −*z* + 3/2.

In absence of classical hydrogen bonds between coformers, Oxyma-B molecules are engaged in weak noncovalent interactions with neighboring Oxyma-B units (Figure 1). The packing is consolidated via a T-shaped lone pair···π interaction with a distance between the oxygen atom and the centroid equal to 2.87 Å, where the carbonyl oxygen aligns perpendicularly above the electron-deficient ring, as well as ancillary CH···O interactions that further strengthens the packing. These interactions collectively support the molecular assembly and the three-dimensional packing.

**Figure 1:**
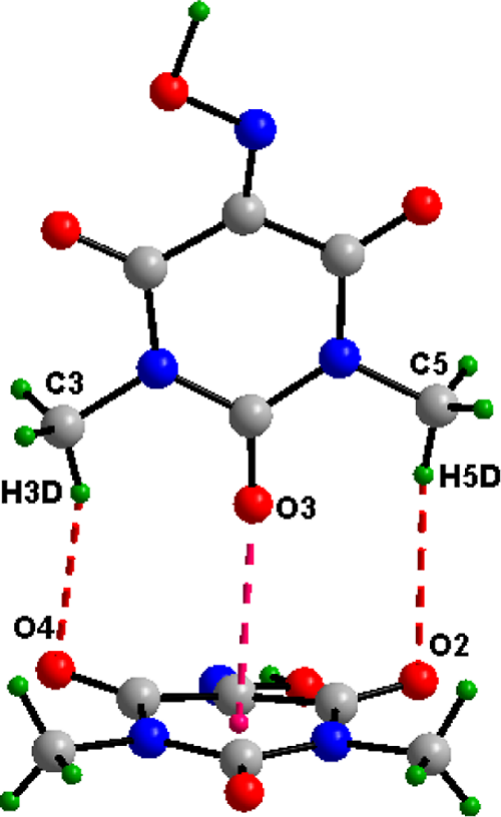
Noncovalent interactions between Oxyma-B molecules in cocrystal I showing a T-shaped O···π contact (2.87 Å) and ancillary H-bonding interactions.

Figure 2 displays a close-up view where the 6-methylquinoline pyridine ring is engaged in face-to-face stacking with the Oxyma-B molecule. The resulting packing is illustrated in Figure 3, highlighting the stacking between the 6-methylquinoline and Oxyma-B rings with distance between centroids of 3.67 Å, and as shown in the same figure, a rich network of NCIs including hydrogen bonds and lone pair···π, which interconnect the molecules throughout the unit cell, generating a rigid and cohesive supramolecular framework.

**Figure 2:**
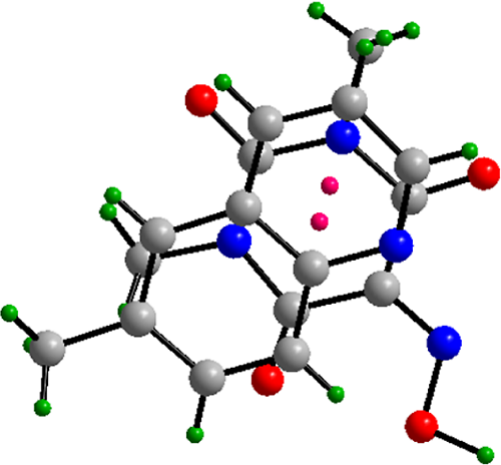
π-Stacking between 6-methylquinoline and Oxyma-B in cocrystal I.

**Figure 3:**
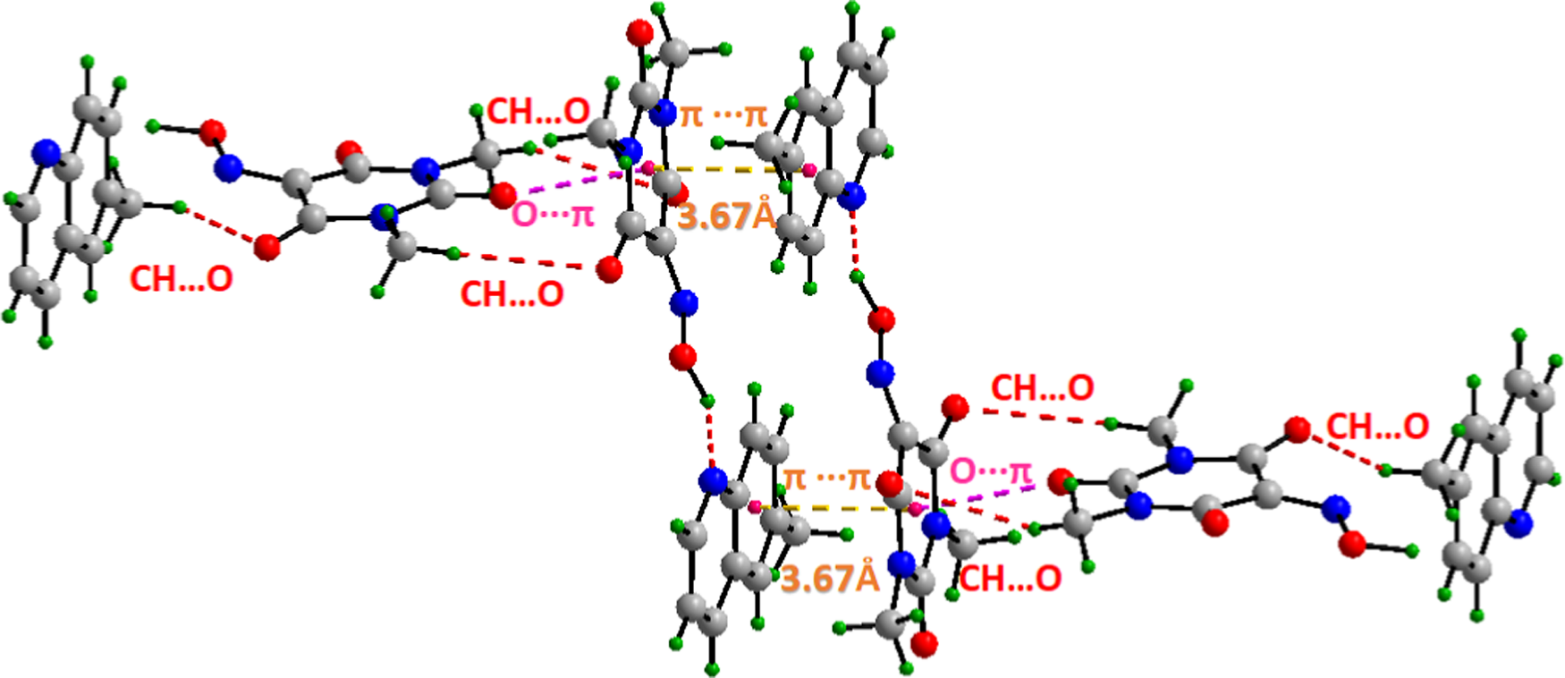
Detailed view of all noncovalent interactions network in 6-methylquinoline/Oxyma-B cocrystal I including HB, lone pair···π and π-stacking interactions between both components.

A closer look to the intermolecular contacts allows the detection of two simultaneous *n* → π* interactions (Figure 4). In particular, the oxygen atom O3 of the donor group approaches the acceptor carbon atoms C2 and C6 with a specific orientation wherein the O–C–O angle is 104° and 106°, respectively (Table 3), very close to which is known as the Bürgi–Dunitz trajectory,^33^ where the value of the angle of approach has been deduced as 105 ± 5° from both crystal structures and computational analysis.^34^

**Figure 4:**
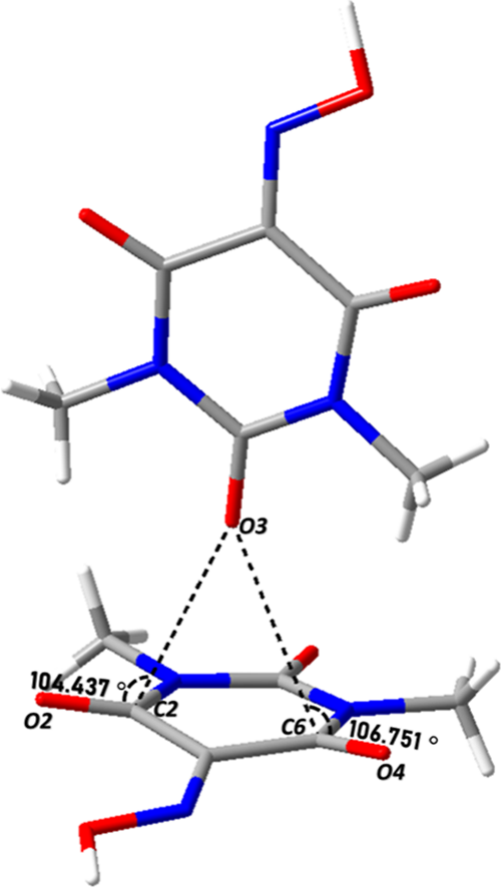
*n* → π* interactions between Oxyma-B molecules in cocrystal I.

**Table 3:** Geometric Parameters of the *n* → π* Interactions between Oxyma-B Molecules in Cocrystal I

donor atom	acceptor atom	O···C (Å)	O···C=O (°)
O3	C2	3.013 (2)	104.5 (1)
O3	C6	3.087 (2)	106.8 (1)

Hirshfeld surface (HS) analysis and corresponding fingerprint plots were performed using Crystal Explorer software.^35,36^ This analysis offers both qualitative and quantitative assessments of the NCIs responsible for the molecular packing observed in the crystal structure. As shown in Figure 5a, the HS mapped over the *d*_norm_ mode reveals prominent red areas indicative of close contacts surrounding H-bonding donor and acceptor. Red patches correspond primarily to significant hydrogen bonds, with a combined H···O/O···H interaction contribution of 24.1%, which also appear as sharp peaks in the associated 2D fingerprint plots (Figure S3). Figure 5b shows the HS mapped over the shape index mode, which is especially informative for visualizing π···π interactions. The alternating red and blue triangular patterns resembling “bow-tie” across the rings of both Oxyma-B and 6-methylquinoline confirm the existence of significant π···π stacking, the blue triangles reflect the convex zones and indicate the presence of electron-rich rings inside the surface, while the red triangles reflect the concave zones resulting from the π-stacking above the surface.^37,38^ This interaction is quantitatively supported by the C···C contacts in the fingerprint plots, accounting for 5.6% of the total surface. Further insight is gained from the fingerprint plots, where CH···π interactions are also observed, represented by C···H/H···C contacts that account for 11.4% of the total surface, characterized by well-defined spikes. Finally, the contribution of lone pair···π interactions has also been detected, which is supported by the presence of C···O/O···C (3.8%) contacts. These are attributed to O···π interactions, involving the lone pairs of oxygen atoms interacting with adjacent π systems of oxyma-B molecules.^39^

**Figure 5:**
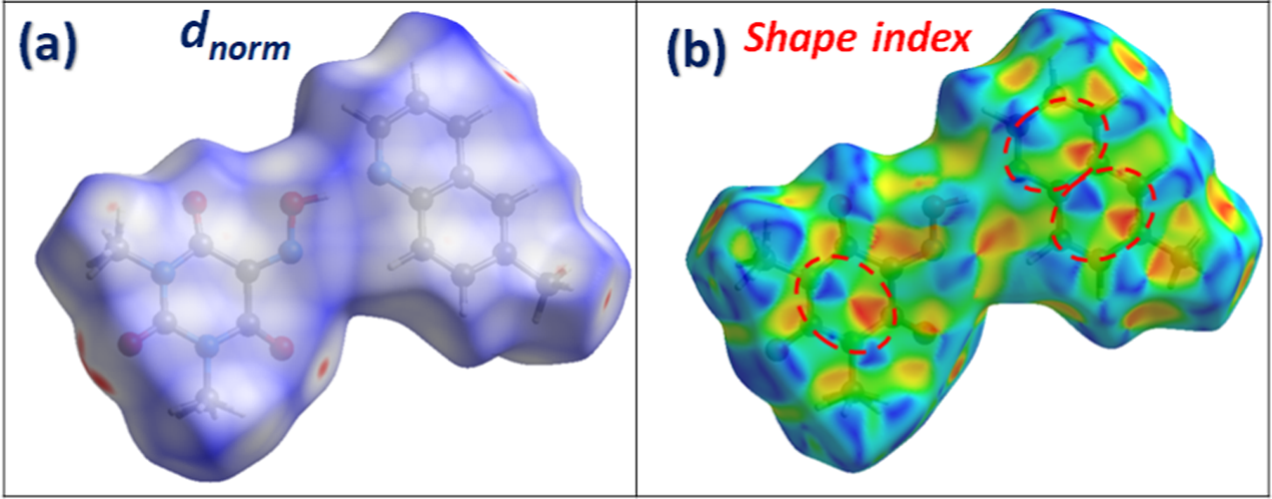
Hirshfeld surfaces analysis of 6-methylquinoline/Oxyma-B cocrystal in *d*_norm_ (a) and shape index (b) mode.

#### 3.1.2 2,3,5,6-Tetramethylpyrazine/Oxyma-B Cocrystal (II)

2,3,5,6-Tetramethylpyrazine/Oxyma-B cocrystal (II), depicted in Figure S4, crystallizes in the monoclinic crystal system with space group *P*2_1_/*n*, comprising one independent molecule of Oxyma-B and half molecule of 2,3,5,6-tetramethylpyrazine in the asymmetric unit. Oxyma-B molecule exhibits positional disorder, with the major conformation refined to an occupancy of 72%, while the minor disordered component designated with the suffix “A” in the atom labels accounts for the remaining 28%.

As illustrated in Figure S5, both forms of Oxyma-B participate in OH···N and CH···O hydrogen bonds, contributing to the formation of a robust supramolecular framework. The geometric parameters of the H-Bonds in cocrystal II are detailed in Table 4.

**Table 4:** Hydrogen-Bonds Geometry in Cocrystal II (Å, °)^a^

D—H···A	D—H (Å)	H···A (Å)	D···A (Å)	D—H···A (°)
C3N—H3NA···O1A	0.98	1.817	2.731 (6)	154
O1A—H1A···N1N	1.0 (1)	1.7 (1)	2.729 (6)	172 (10)
C5—H5A···O6^i^	0.98	2.682	3.661	176.56
C3—H3A···O6^ii^	0.979 (1)	2.728 (2)	3.647 (4)	168.62
C5—H5C···O2^ii^	2.980 (1)	2.933 (3)	3.862 (4)	158.56
C5—H5NA···O1	0.98	2.313	3.158 (4)	144
O1—H1···N1N	1.00 (5)	1.69 (5)	2.688 (3)	170 (5)
C3N—H3NA···N1^i^	0.98	2.734 (2)	3.655 (2)	156.76

a Symmetry code: (i) *x*, *y* + 1, *z*; (ii) −*x* + 1/2, *y* + 1/2, 3/2 – *z*.

To further explore the supramolecular organization, the crystal packing was analyzed using the major disorder component of the Oxyma-B molecule. As illustrated in Figure S6a, the packing along the ( a⃗,c⃗) plane reveals a ribbon arrangement formed by alternating Oxyma-B and 2,3,5,6-tetramethylpyrazine molecules. The cohesion within the structure is ensured by a network of OH···N and CH···N hydrogen bonds. A closer view provided in Figure S6 b highlights how these hydrogen bonds give rise to *R*_2_^2^(7) supramolecular synthons, contributing to the organization of the crystal lattice.

To better visualize the spatial distribution, Oxyma-B molecules were projected separately (Figure 6). The resulting framework highlights a dense network sustained by multiple NCIs, including ancillary CH···O hydrogen bonds and lone pair···π (O···π) interactions (2.76 Å), Collectively, these NCIs ensure the interconnection of Oxyma-B units throughout the crystal structure.

**Figure 6:**
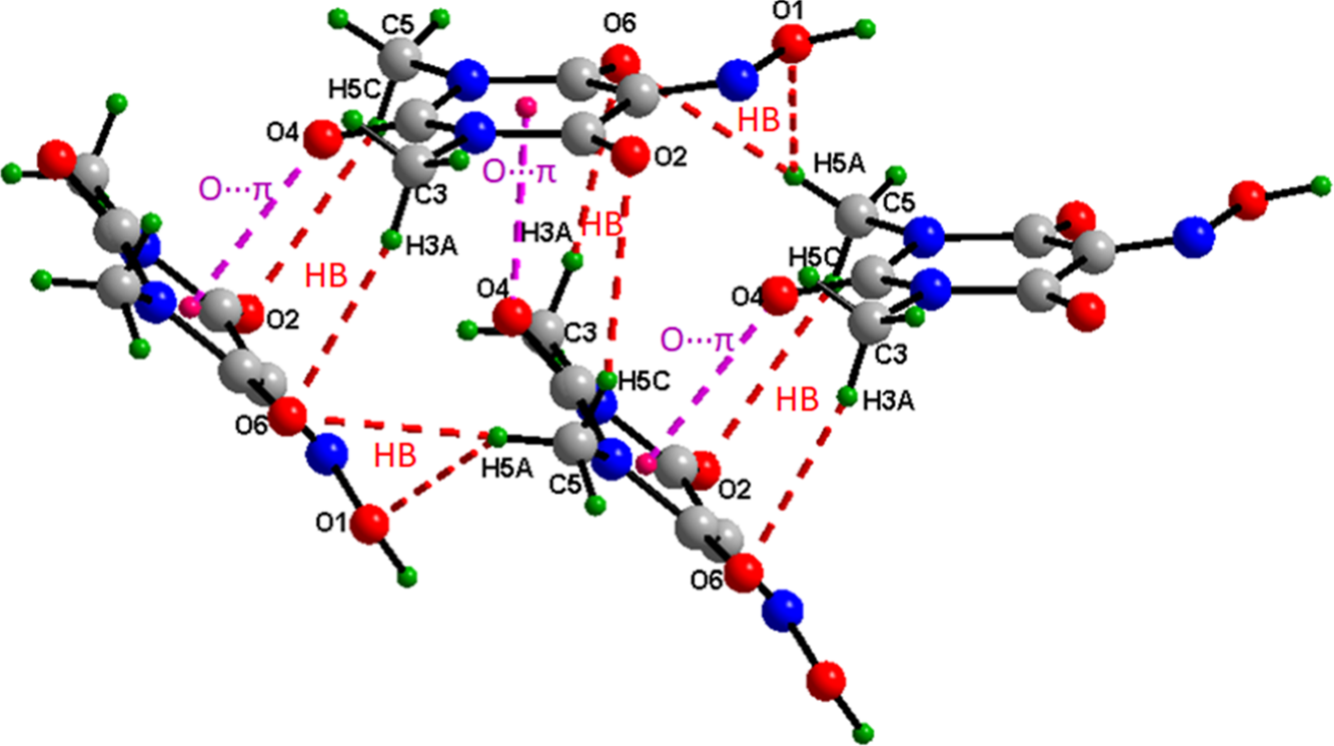
Projection of Oxyma-B molecules within the crystal packing of cocrystal II, showing the network of CH···O and O···π interactions.

In the case of cocrystal II, a closer examination of the molecular packing reveals three simultaneous *n* → π* interactions occurring between Oxyma-B molecules. Specifically, the oxygen atom O4 of one Oxyma-B (major disorder component) acts as a lone pair donor, approaching the electrophilic carbon atoms C2, C4 and C6 of neighboring carbonyl groups (Figure 7a). These contacts are characterized by O···C separations that fall within the accepted range for such interactions, and by approach angles of 104°, 105° and 120°, respectively. For the minor disorder component, the corresponding interactions involve O4A approaching C2A, C4A, and C6A (Figure 7b), with approach angles of 127°, 102°, and 105°, respectively (Table 5).

**Figure 7:**
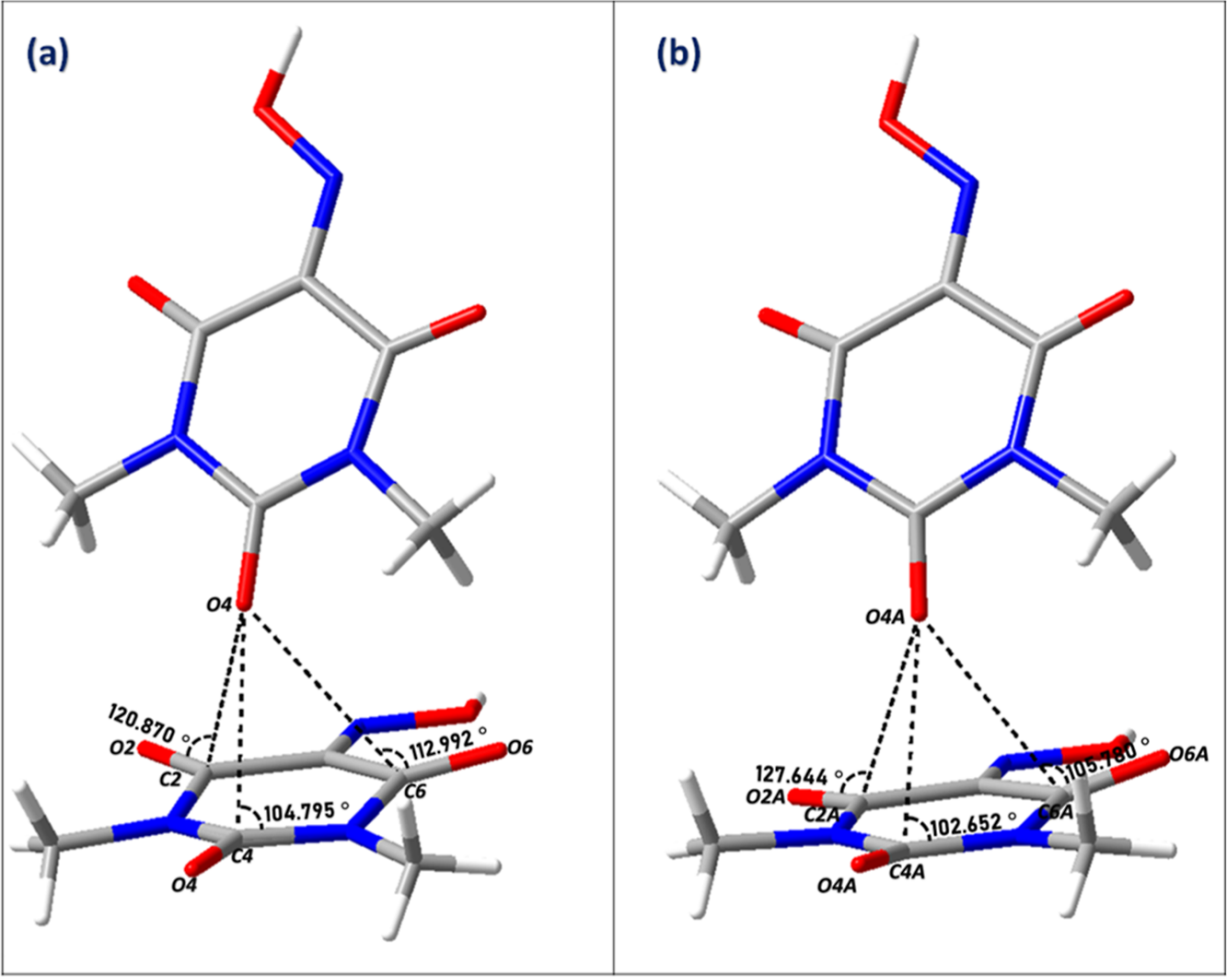
*n* → π* interactions between Oxyma-B molecules in cocrystal II: (a) interactions involving the major disorder component; (b) corresponding interactions for the minor disorder component.

**Table 5:** Geometric Parameters of the *n* → π* Interactions between Oxyma-B Molecules in Cocrystal II

donor atom	acceptor atom	O···C (Å)	O···C=O (°)
O4	C4	2.87	105
O4	C6	3.14	113
O4	C2	3.12	121
O4A	C4A	2.82	103
O4A	C6A	3.02	106
O4A	C2A	3.20	128

The main difference between cocrystal I and cocrystal II in terms of *n* → π* interactions is that in the former they occur only with the two carbon atoms adjacent to the oxime group (C2 and C6), while in cocrystal II they occur with all three carbon atoms (C2, C4 and C6), Figure 8. This makes the distance between the donor oxygen and the oxyme carbon (C1) longer in II than in I with a direct impact on the higher loss of planarity of the oxime group in I with respect to II. Thus, the angle formed by N1, the calculated centroid and C4 is 169.6° and 173.3° in I and II respectively (not shown).

**Figure 8:**
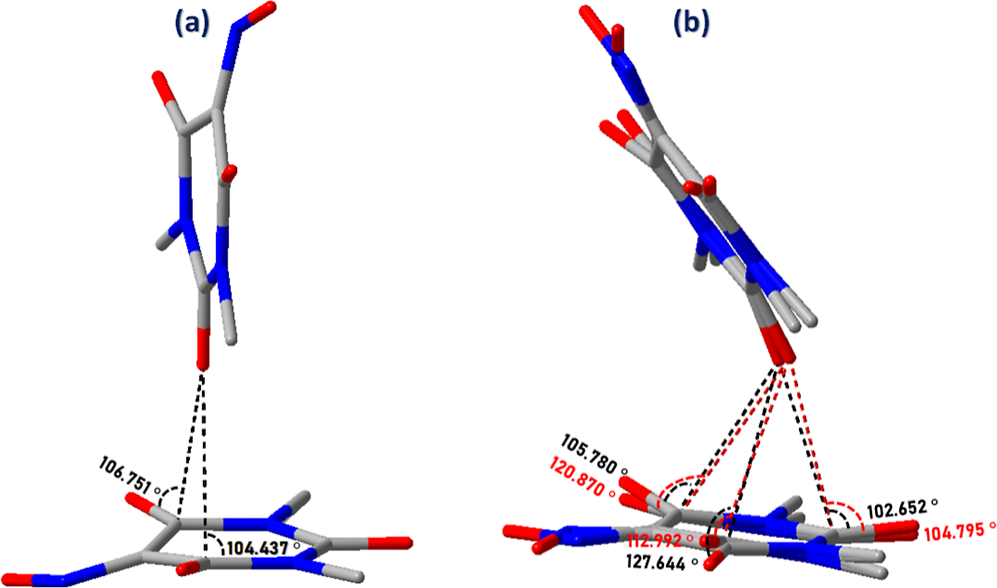
Comparison of *n* → π* interactions in Oxyma-B molecules between (a) cocrystal I and (b) cocrystal II. Red dashed lines in (b) represent interactions in the major disordered component, while black dashed lines correspond to the minor component.

This can be rationalized from a repulsion point of view since the lone-pair donation in oximes to the α-carbon by oxygen decreases the electrophilicity relative to carbonyls or imines (Figure 9). Thus, in order to reduce repulsion between the donor oxygen and the α-carbon (C1) the ring bends in the direction opposite to the *n* → π* interaction more in I than in II.

**Figure 9:**
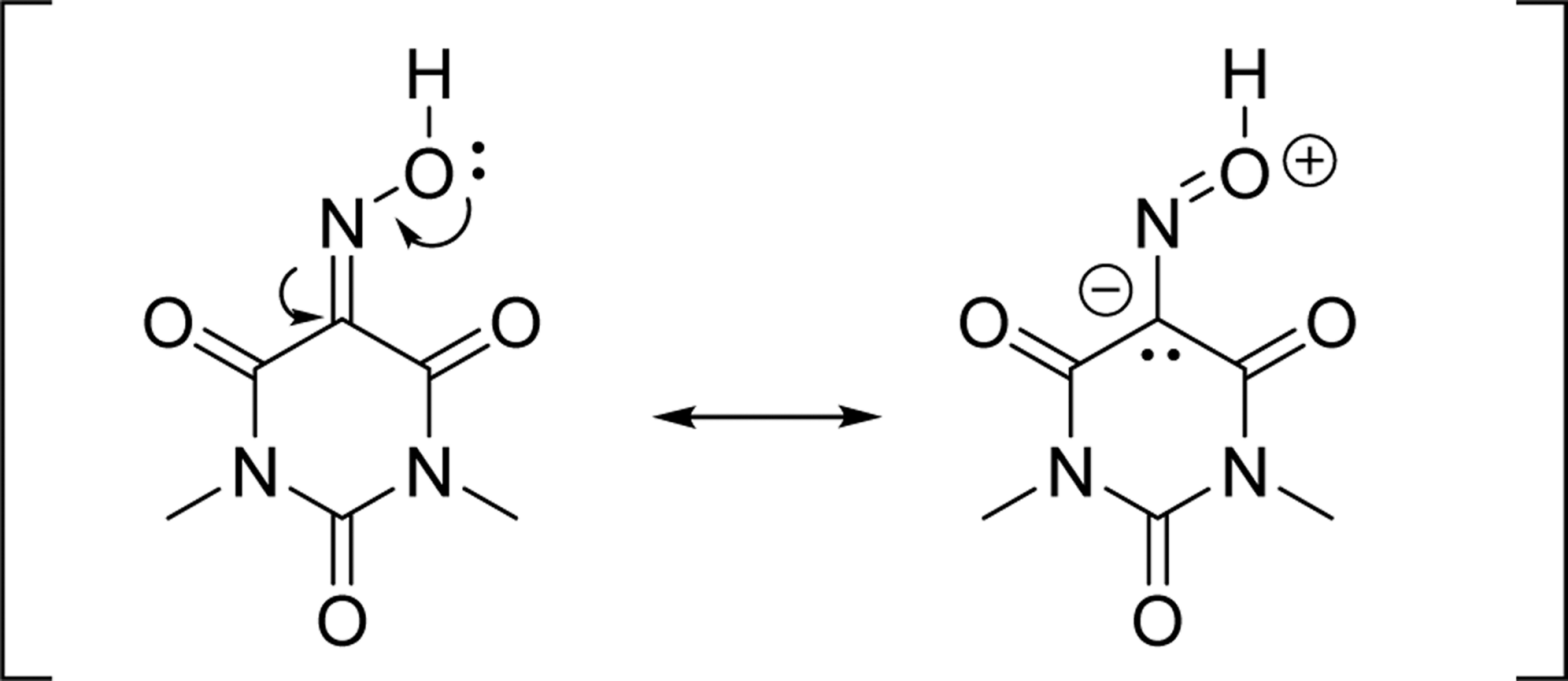
Resonance forms of Oxyma B.

As shown in Figure 10a, the Hirshfeld surface (HS) mapped over the *d*_norm_ mode reveals distinct red regions, which correspond predominantly to OH···N and CH···O hydrogen bonds. These interactions are quantitatively reflected in the two-dimensional fingerprint plots (Figure S7), where H···N/N···H and H···O/O···H contacts collectively account for 34.4% of the total surface area highlighting the central role of hydrogen bonding in the stabilization of the crystal structure. Notably, the fingerprint plots also indicate the presence of the lone pair···π interactions, shown by the C···O/O···C (5.4%) contacts. The HS mapped over the shape index mode (Figure 10b) reveals alternating red and blue triangular motifs on the rings of both Oxyma-B and 2,3,5,6-tetramethylpyrazine, representing the impact of π-stacking interactions. This is corroborated by the presence of C···C contacts in the fingerprint plots, accounting for 2.9% of the surface. Additionally, CH···π interactions are also detected via the C···H/H···C contacts, contributing 2.2%. Together, these interactions illustrate the varied nature of the NCIs that govern supramolecular assembly in the cocrystal II.

**Figure 10:**
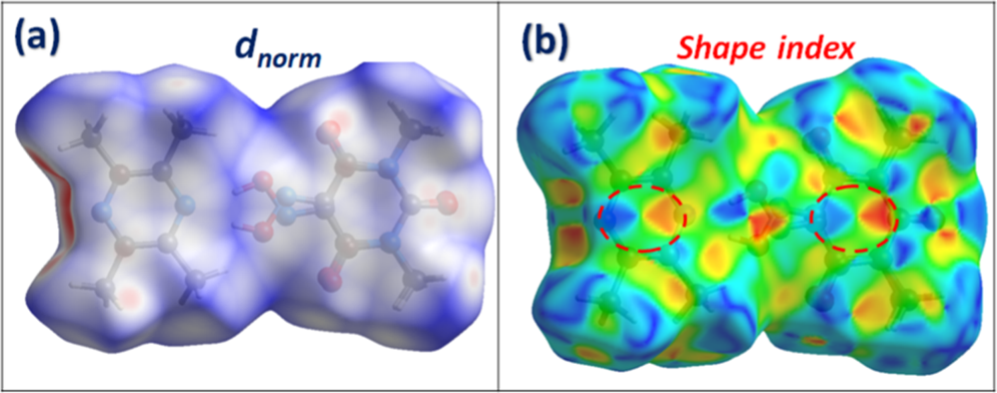
Hirshfeld surfaces of cocrystal II: (a) d_norm_ and (b) shape index map.

#### 3.1.3 1,10-Phenanthroline/Oxyma-B Cocrystal (III)

1,10-phenanthroline/Oxyma-B cocrystal (III), crystallizes in the orthorhombic crystal system with space group *P*2_1_2_1_2_1_. The asymmetric unit comprises one independent molecule of each component, Figure S8.

The crystal packing of 1,10-phenanthroline/Oxyma-B cocrystal (III) is illustrated in Figure S9. The projection along the ( a⃗,c⃗) plane reveals that the molecules are arranged in a distinct zigzag pattern, propagating along *y* = 0 and *y* = 1/2 planes. These chains are interconnected via intermolecular CH···O hydrogen bonds, while intrachain stability is reinforced by both OH···N and additional CH···O interactions, forming a robust hydrogen-bonding network. The geometric parameters of these hydrogen bonds are detailed in Table 6.

**Table 6:** Hydrogen-Bonds Geometry in Co-Crystal I (Å, °)^a^

D—H···A	D—H (Å)	H···A (Å)	D···A (Å)	D—H···A (°)
O1—H1···N1N^i^	1.08 (4)	1.64 (4)	2.700 (3)	165 (3)
C3N—H3N···O4^ii^	0.95	2.49	3.203 (4)	132
O1—H1···N10N^i^	1.08 (4)	2.54 (4)	3.101 (3)	111 (2)
C5—H5B···O6^iii^	0.98	2.68	3.594 (4)	155
C5—H5B···O1^iii^	0.98	2.53	3.223 (4)	128
C6N—H6N···O6^iv^	0.95	2.35	3.215 (4)	151
C5N—H5N···O2^v^	0.95	2.40	3.285 (4)	155

a Symmetry codes: (i) −*x* + 2, *y* + 1/2, −*z* + 1/2; (ii) −*x* + 3/2, −*y*, *z* – 1/2; (iii) −*x* + 3/2, −*y* + 1, *z* + 1/2; (iv) −*x* + 3/2, −*y* + 1, *z* – 1/2; (v) *x* −1/2, −*y* + 1/2, −*z* + 1.

The hydrogen-bonding architecture in cocrystal (III) is characterized by the formation of massive supramolecular motifs. As shown in Figure 11, the cooperative engagement of CH···O and OH···N hydrogen bonds organizes the molecules into two supramolecular synthons *R*_4_^4^(20) and *R*_5_^5^(24) with the supported of two additional smaller motifs of type *R*_1_^2^(5) and *R*_1_^2^(6). These supramolecular synthons are interlinked to construct a highly ordered, multidimensional hydrogen-bonding network.

**Figure 11:**
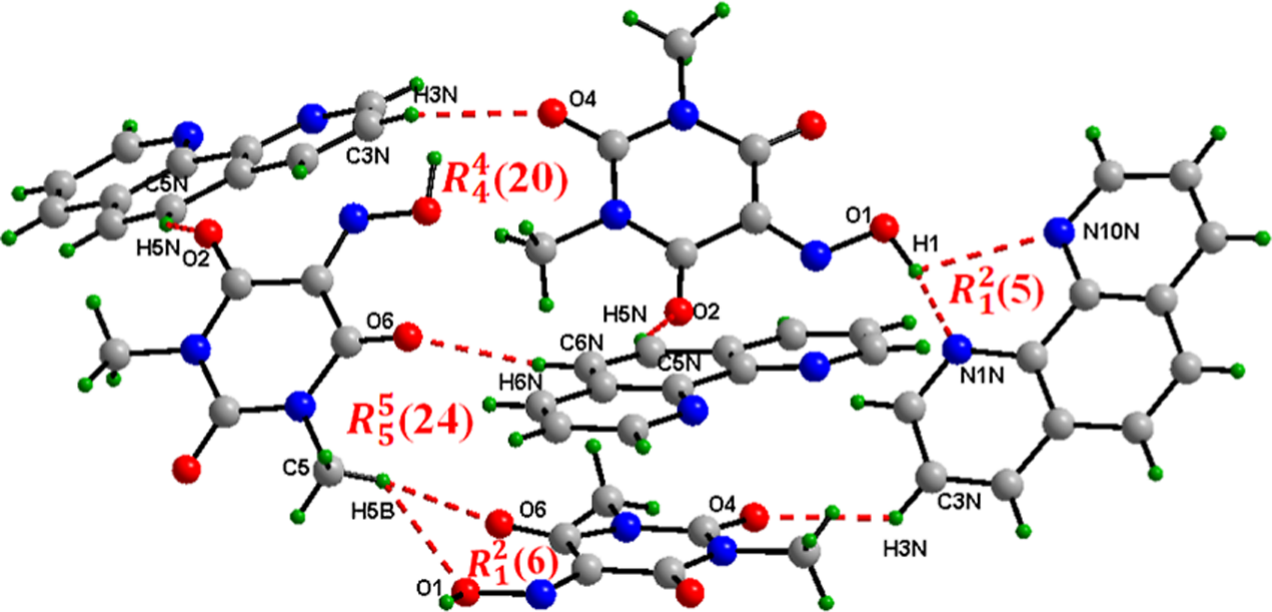
*R*_4_^4^(20) and *R*_5_^5^(26) supramolecular synthons in 1,10-phenanthroline/Oxyma-B cocrystal (III).

In Figure S10a, the projection along the ( a⃗,b⃗) plane reveals molecular chains aligned in a parallel arrangement, held together by hydrogen bonds. Figure S10b presents a close-up view highlighting the parallel packing arrangement, where 1,10-phenanthroline engages in face-to-face stacking with Oxyma-B molecules. As illustrated in Figure 12, the supramolecular architecture of the cocrystal III is further stabilized by a network of noncovalent interactions involving π-systems. π···π interactions are observed between 1,10-phenanthroline and Oxyma-B, with centroid-to-centroid distances of 3.40 Å and 3.46 Å. Additionally, CH···π contacts are established between the methyl group of Oxyma-B molecules and the phenanthroline rings, with hydrogen to centroid distances of 3.36 Å and 3.48 Å.

**Figure 12:**
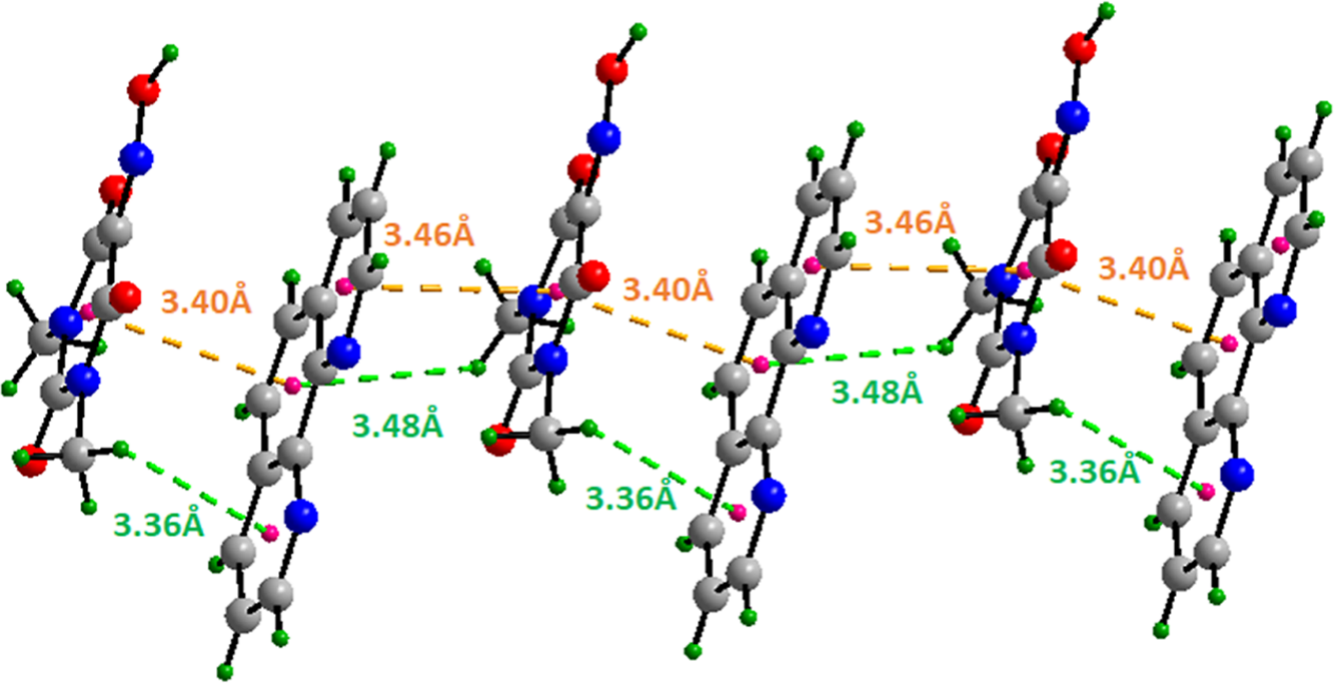
View of π-stacking (3.40 and 3.46 Å) and CH···π interactions (3.36 and 3.48 Å) between 1,10-phenanthroline and Oxyma-B.

Figure [Fig fig1]3a presents the HS mapped over the d_norm_ mode of the cocrystal III, highlighting prominent red spots corresponding to significant close contacts, notably H···N/N···H and H···O/O···H interactions. These contacts are quantified at 15.4% and 27.1%, respectively, and are associated with sharp, elongated spikes in the 2D fingerprint plots (Figure S11), attributed to OH···N and CH···O hydrogen bonds. The HS mapped over the shape index mode (Figure 13b) displays alternating red/blue “bow-tie” motifs on the ring surfaces of both Oxyma-B and 1,10-phenanthroline, confirming the presence of π-stacking interactions, further supported by a 3.8% contribution from C···C contacts. A substantial presence of C···H/H···C contacts (11.7%) indicates also the existence of CH···π interactions. In contrast, the lower percentages of C···O/O···C (2.1%) and C···N/N···C (1.9%) contacts, compared to cocrystals I and II, explain the absence of lone pair···π contacts, distinguishing this cocrystal from the previous ones.

**Figure 13:**
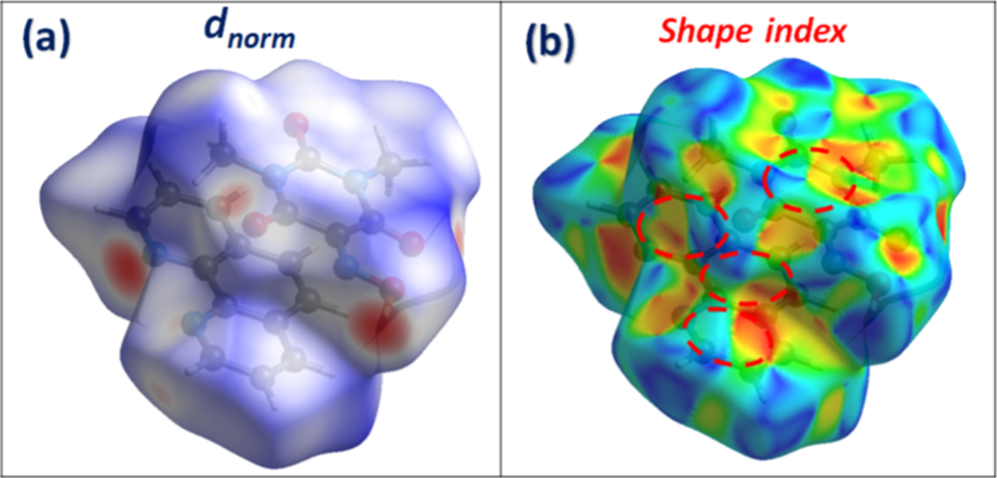
Hirshfeld surfaces of Co-crystal III: (a) d_norm_ and (b) shape index map.

Interestingly, no *n* → π* interactions between the oxyma B molecules have been observed in this cocrystal and the angle formed by N1, the calculated centroid and C4 is the highest in the three cocrystals (174.4°), which is consistent with the reasoning explained above.

#### 3.1.4 DFT Calculations

The DFT study focuses on analyzing the hydrogen-bonding interactions between the coformers, as well as the cooperative effects involving hydrogen bonding, lone pair···π (LP···π), and π-stacking interactions. We began by computing the molecular electrostatic potential (MEP) surfaces of the individual coformers and selected cocrystallized molecular pairs to assess how the electrostatic distribution evolves upon dimerization, particularly in regions associated with intermolecular interactions.

Figure 14 (top) displays the MEP surface of Oxyma-B. The most positive region is located at the hydroxyl hydrogen (+59.0 kcal/mol), while the most negative region corresponds to a carbonyl oxygen atom adjacent to the oxime group (−36.1 kcal/mol). Notably, the oxygen atom involved in the previously discussed O···π interaction shows a less negative potential (−26.0 kcal/mol). The MEP over the center of the ring is +28.6 kcal/mol, highlighting the electron-deficient character of the six-membered ring. The MEP surfaces of the coformer bases, 6-methylquinoline, 2,3,5,6-tetramethylpyrazine, and 1,10-phenanthroline, are presented in Figure 14b–d. In all three molecules, the MEP minimum is localized at the nitrogen atom, ranging from −29.6 kcal/mol in 2,3,5,6-tetramethylpyrazine to −55.7 kcal/mol in 1,10-phenanthroline. The MEP maxima are associated with hydrogen atoms. Aromatic hydrogens exhibit higher positive values (+20.4 and +22.0 kcal/mol in 6-methylquinoline and 1,10-phenanthroline, respectively) compared to aliphatic hydrogens (+14.2 kcal/mol in 2,3,5,6-tetramethylpyrazine). Across all cases, the MEP values over the aromatic rings are moderately negative (ranging from −3.5 to −10.0 kcal/mol), with 6-methylquinoline showing the most pronounced values. This MEP analysis supports the formation of OH···N hydrogen bonds between the oxime proton of Oxyma-B and the nitrogen atoms of the bases, as observed in the X-ray structures. To further explore this interaction, we optimized the hydrogen-bonded dimers and recomputed the MEP surfaces to evaluate how electrostatic features shift upon complex formation. Moreover, in the case of 6-methylquinoline, we also computed the π-stacked dimer to examine how the π-acidity of the Oxyma-B ring is affected by stacking. As shown in Figure 14e, the MEP over the pyrimidine ring is reduced by approximately 4 kcal/mol, while the oxygen atoms become more nucleophilic (i.e., exhibit more negative MEP values). This result suggests that π-stacking has a dual effect on Oxyma-B’s capacity to engage in LP···π interactions: although the π-acidity of the pyrimidine ring decreases, the lone pair donor ability of the oxygen atoms is enhanced.

**Figure 14:**
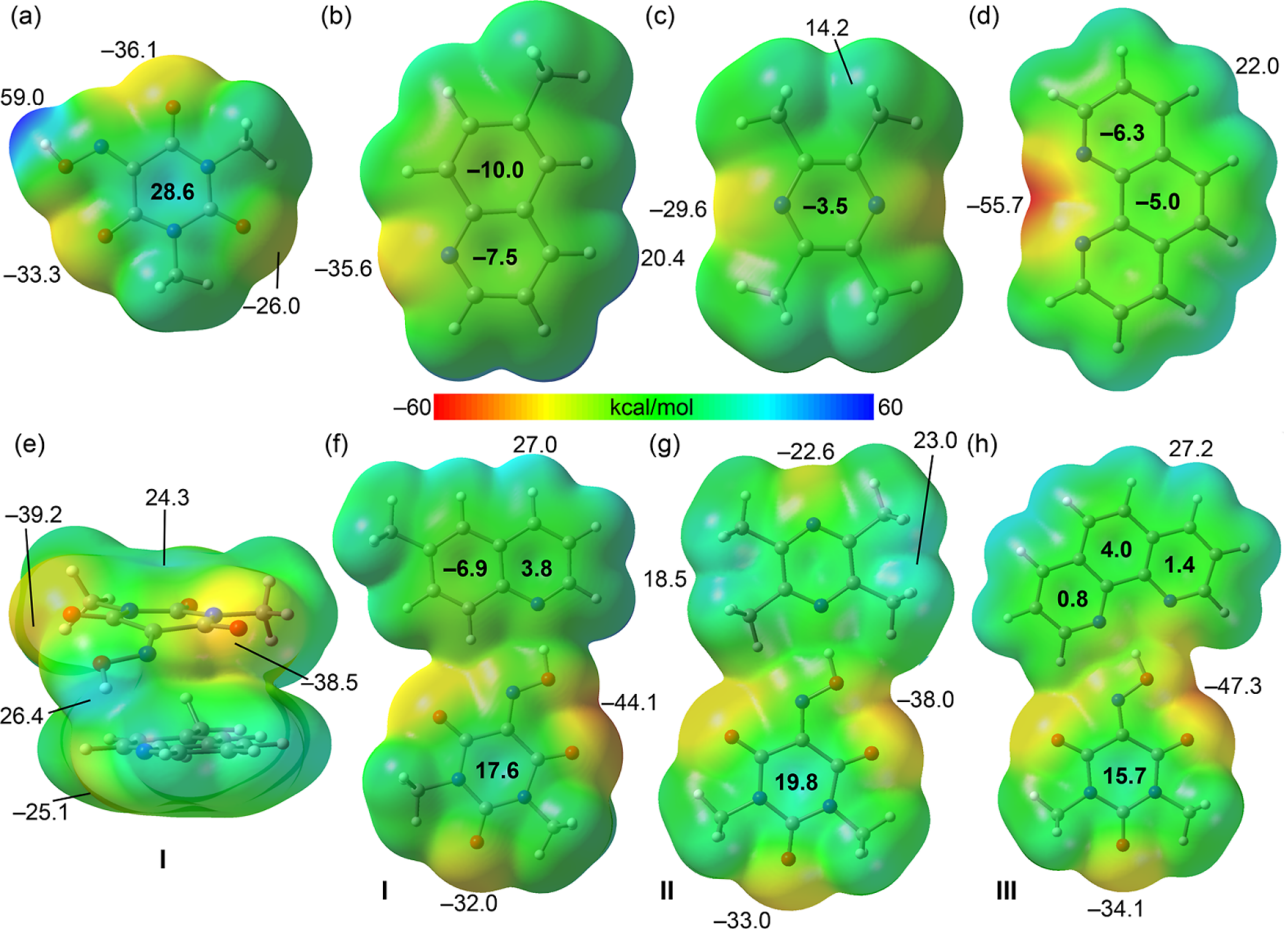
MEP surfaces of Oxyma-B (a), 6-methylquinoline (b), 2,3,5,6-tetramethylpyrazine (c), 1,10-phenanthroline (d), π-stacking cocrystallized molecular pair of I (e), H-bond cocrystallized molecular pairs of I (f), II (g) and III (h). Isovalue 0.001 au Energies at selected points in kcal/mol.

Figure 14f shows the MEP surface of the hydrogen-bonded cocrystallized molecular pair formed between Oxyma-B and 6-methylquinoline. Upon complexation, the MEP over the pyrimidine ring is further reduced, and the MEP value at the oxygen atom opposite to the oxime group becomes more negative (−32.0 kcal/mol). A similar trend is observed for the hydrogen-bonded cocrystallized molecular pairs of Oxyma-B with 2,3,5,6-tetramethylpyrazine and 1,10-phenanthroline. Notably, the most pronounced effect is seen with 1,10-phenanthroline, the best hydrogen bond acceptor among the three, where the MEP over the pyrazine unit decreases to +15.7 kcal/mol. This finding correlates with the absence of LP···π interactions in this complex. Additionally, the MEP values over the phenanthroline ring shift from negative to slightly positive values, indicating a diminished electrostatic repulsion. This shift facilitates π-stacking interactions, as the reduced polarity enhances overlap between the π-systems of the interacting species.

Next, we analyzed the energetics and topological features of the optimized hydrogen-bonded cocrystallized molecular pairs of Oxyma-B with 6-methylquinoline, 2,3,5,6-tetramethylpyrazine, and 1,10-phenanthroline using dimerization energy calculations and the quantum theory of atoms in molecules (QTAIM). The results, presented in Figure 15 reveal significant dimerization energies ranging from −14.8 kcal/mol for cocrystal II to −18.2 kcal/mol for cocrystal III. For cocrystal I, the calculated dimerization energy is −16.0 kcal/mol. These large binding energies confirm the strong nature of the hydrogen bonds and underscore their importance in stabilizing the solid-state architectures of the cocrystals.

**Figure 15:**
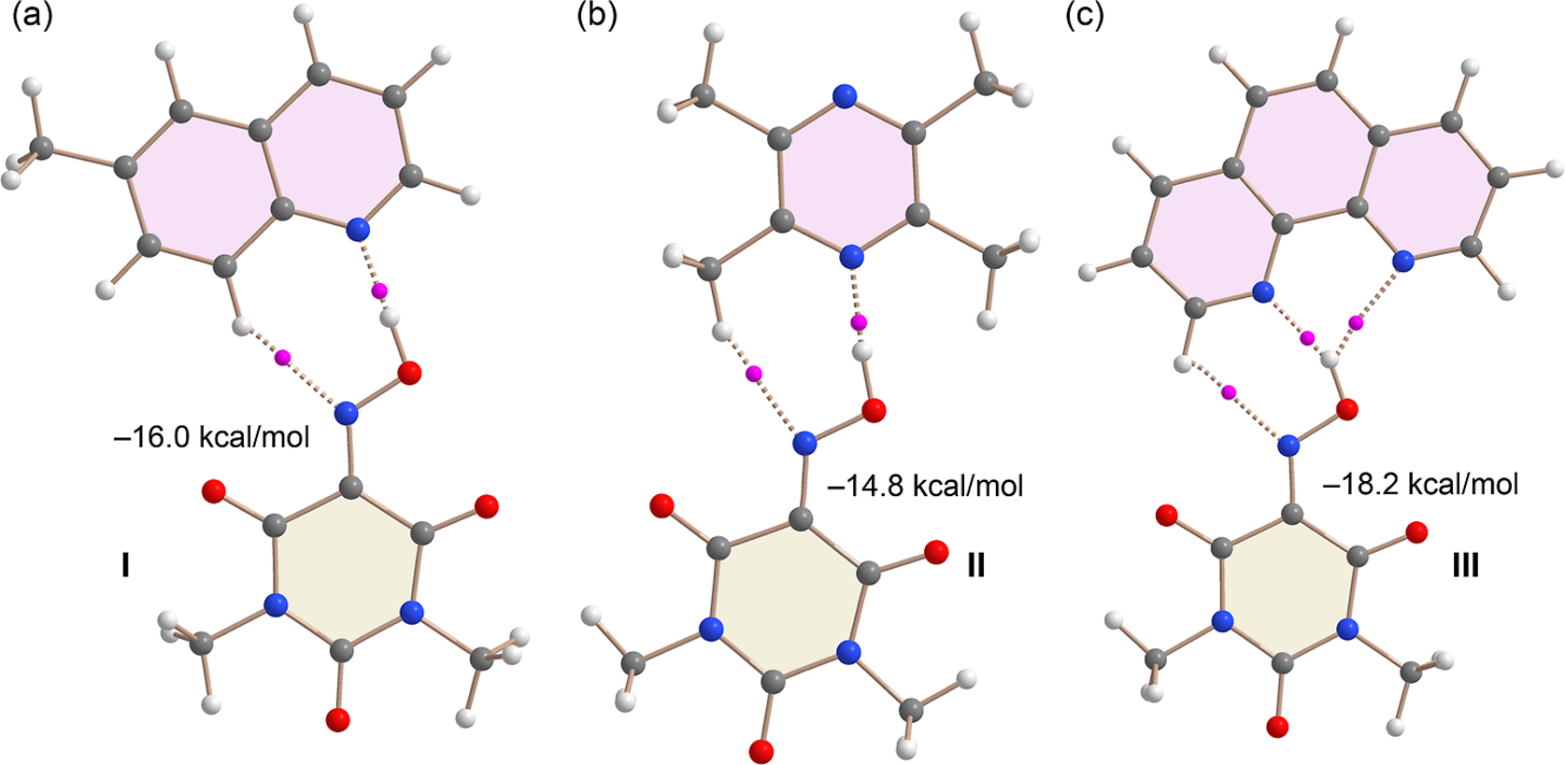
QTAIM analysis of the HB optimized cocrystallized molecular pairs of I (a), II (b) and III (c). Only intermolecular BCP (as fuchsia spheres) are indicated. Th bond paths are indicated as dashed bonds. The dimerization energies are indicated at the PBE0-D3/def2-TZVP level of theory.

The QTAIM analysis reveals that all cocrystallized molecular pairs adopt classical *R*_2_^2^(7) hydrogen-bonding motifs, each comprising a strong OH···N hydrogen bond accompanied by a secondary CH···N interaction involving a single C–H donor. These hydrogen bonds are characterized by the presence of bond critical points (BCPs, shown as fuchsia spheres) and bond paths (represented as dashed lines) connecting the hydrogen atom to the nitrogen acceptor atom. Notably, in cocrystal III, an additional OH···N interaction is observed. Although this hydrogen bond is less directional, it contributes to the overall stabilization of the cocrystallized molecular pair and aligns with the higher dimerization energy and the more negative MEP values at the nitrogen atoms involved.

We also investigated the influence of hydrogen bonding and π-stacking on the strength of LP···π interactions in the solid state of cocrystals I and II. To this end, we employed X-ray coordinates and constructed two assemblies for each cocrystal. For cocrystal I, we extracted a representative HB/LP···π/π···π assembly from the solid-state structure (highlighted in Figure 3) and compared the LP···π interaction energies in the presence and absence of the HB and π···π interactions, as illustrated in Figure 16a,b. For each assembly, we also performed QTAIM and NCIplot analyses.

**Figure 16:**
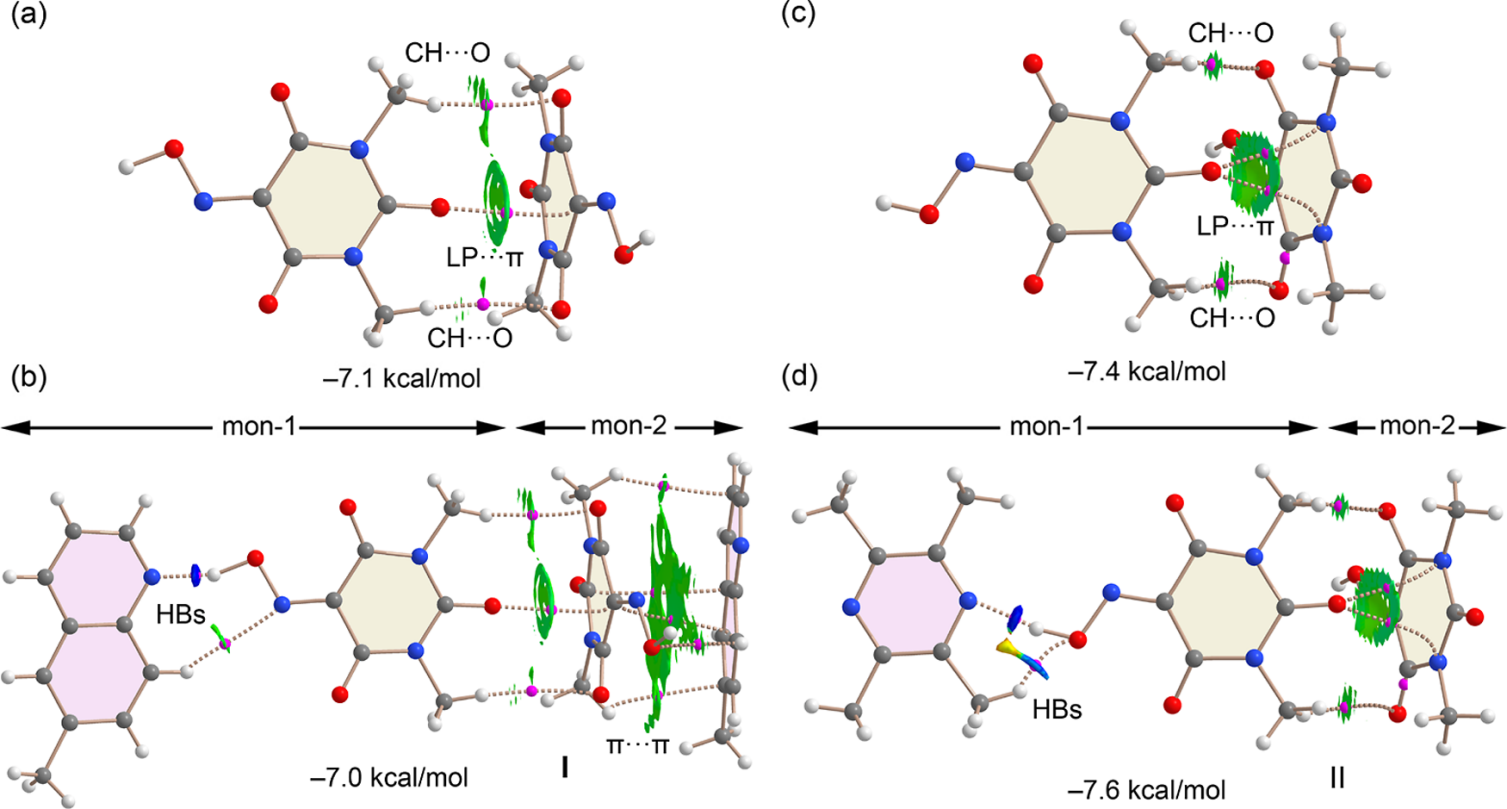
QTAIM and NCIplot analysis of the dimer (a) and tetramer (b) of cocrystal I, and dimer (c) and trimer (b) of cocrystal II. The dimerization energies are indicated. Only intermolecular interactions are represented.

In the LP···π dimer (Figure 16a), the oxygen atom of one Oxyma-B molecule interacts with the pyrimidine ring of an adjacent molecule, evidenced by a BCP and bond path connecting the O atom to a carbon atom in the aromatic ring. The π-nature of this contact is further confirmed by the NCIplot, which shows a broad reduced density gradient (RDG) isosurface enveloping the pyrimidine ring. Additionally, two CH···O interactions involving methyl groups of Oxyma-B are observed. The dimerization energy is moderately strong (−7.1 kcal/mol), arising from the combined LP···π and CH···O interactions. In the more complex HB/LP···π/π···π assembly (Figure 16b), QTAIM analysis reveals several BCPs and bond paths connecting the 6-methylquinoline and Oxyma-B rings, consistent with π-stacking. The interaction energy was computed by treating the system as a dimer, where the monomers are the hydrogen-bonded and π-stacked dimers, as defined in Figure 16b. The resulting interaction energy (−7.0 kcal/mol) is nearly identical to that of the LP···π dimer, suggesting that the strength of the LP···π interaction is unaffected by the additional interactions. This is likely due to a compensating electrostatic effect: while the formation of HB or π-stacked dimers reduces the π-acidity of the pyrimidine ring, it simultaneously enhances the electron-donating ability of the O atom, as indicated by MEP surface analysis.

A similar trend is observed in cocrystal **II**. Here, we examined the LP···π dimer and the HB/LP···π trimer shown in Figure 16c,d. The QTAIM/NCIplot analysis of the LP···π dimer reveals two BCPs and bond paths connecting the O atom to two nitrogen atoms in the pyrimidine ring. The corresponding RDG isosurface between the O atom and the ring is characteristic of π-type interactions. As in cocrystal I, two additional CH···O interactions are observed, and the dimerization energy is similar (−7.4 kcal/mol). In the HB/LP···π trimer (Figure 16d), the interaction energy, computed using the same dimer-based approach, is −7.6 kcal/mol, indicating only a negligible enhancement from the hydrogen bond.

Taken together, the energetic and topological analyses of cocrystals **I** and **II** indicate that the LP···π interaction is essentially unaffected by the presence of hydrogen bonding and π-stacking, likely due to compensatory changes in electrostatic potential.

In Figure 17, we examine how the hydrogen bonding interactions between Oxyma-B and 1,10-phenanthroline in cocrystal **III** influence the strength and nature of the π-stacking interaction. As shown by the MEP surface analysis, hydrogen bonding reduces the MEP values over the phenanthroline ring, thereby diminishing electrostatic repulsion and potentially enhancing π-stacking, where dispersion forces dominate.

**Figure 17:**
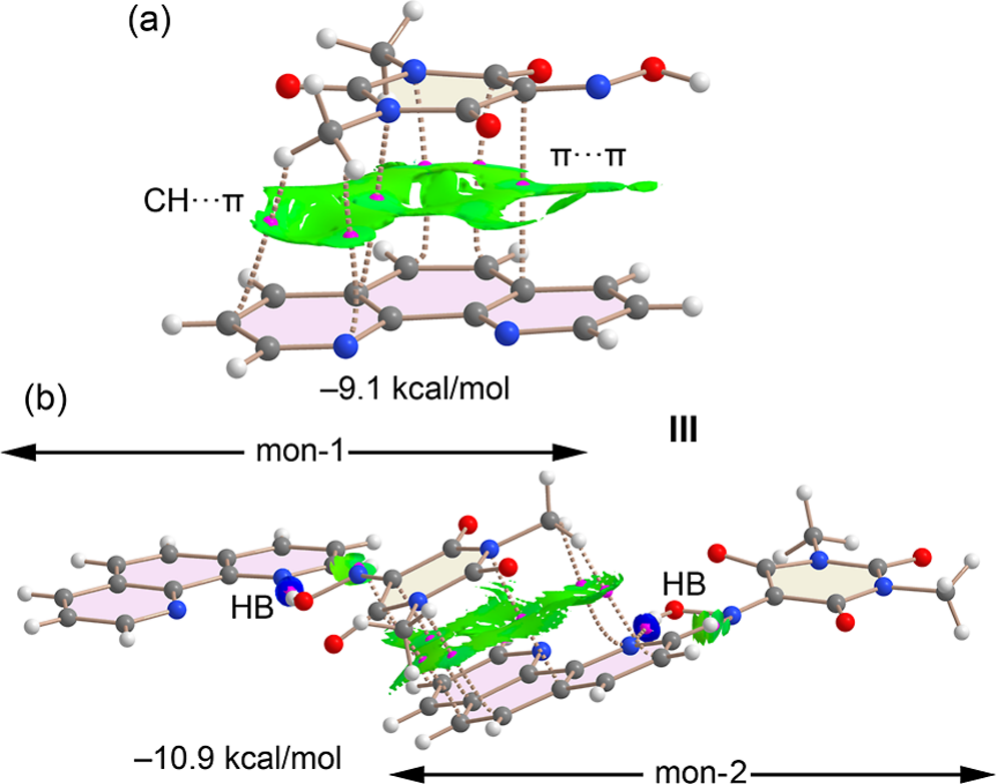
QTAIM and NCIplot analysis of the cocrystallized molecular pair (a) and tetramer (b) of cocrystal III. The dimerization energies are indicated. Only intermolecular interactions are represented.

The isolated π···π cocrystallized molecular pair formed between Oxyma-B and 1,10-phenanthroline displays multiple bond critical points (BCPs) interconnecting the two monomers, along with an extended green RDG isosurface encompassing the entire space between them, indicative of a strong and well-aligned π···π interaction. Additionally, two BCPs and bond paths connect methyl hydrogen atoms of Oxyma-B to carbon atoms of the phenanthroline ring, confirming the presence of complementary CH···π interactions. The computed interaction energy for this π-stacking cocrystallized molecular pair is −9.1 kcal/mol, which is notably stronger than the LP···π interactions observed in cocrystals **I** and **II**.

We further modeled a tetrameric assembly, in which each monomer involved in the π-stacking interaction also engages in hydrogen bonding (Figure 17b). Calculating the interaction energy of this tetramer as a dimer of dimers yields a value of −10.9 kcal/mol, −1.8 kcal/mol more favorable than the isolated cocrystallized molecular pair. This result suggests a favorable cooperative effect between the hydrogen bonding and π-stacking interactions in this system.

As discussed in the structural description and in the rationale behind the differing orientations of the Oxyma-B molecules in the LP···π assemblies of cocrystals I and II, these interactions can be understood as *n* → π* contacts. To support this hypothesis, we performed a natural bond orbital (NBO) analysis, which is particularly useful for probing orbital charge-transfer in donor–acceptor interactions. The NBOs involved in the *n* → π* contacts within the cocrystallized molecular pairs of cocrystals I and II are shown in Figure 18.

**Figure 18:**
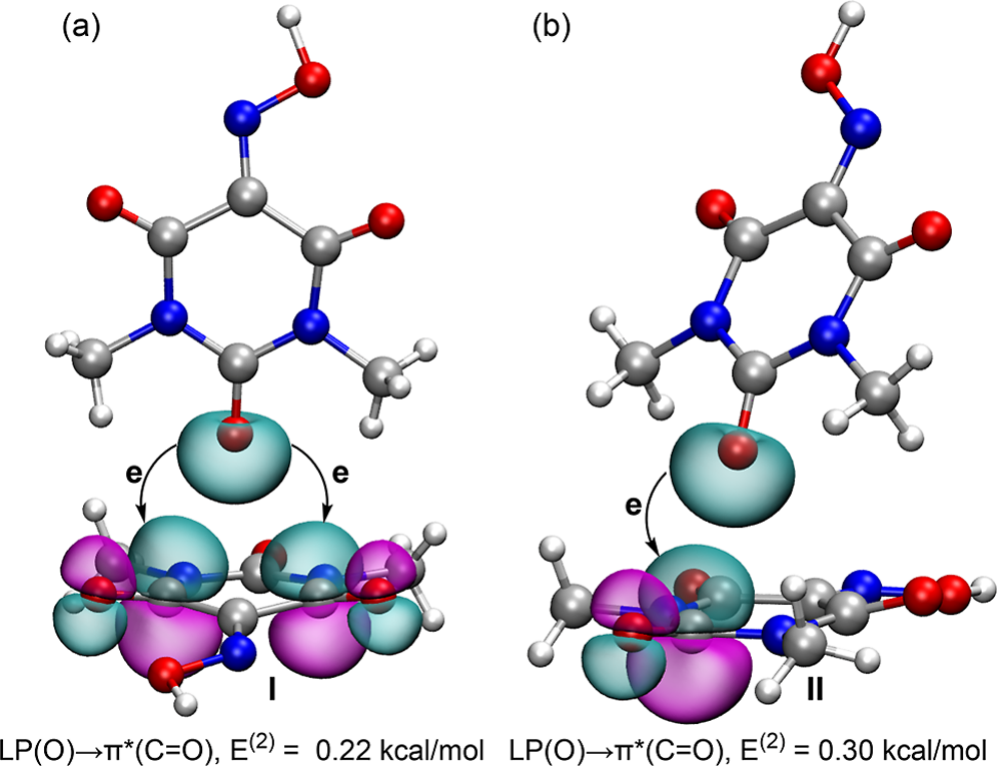
Plots of the NBOs involved in the LP­(O) → π*­(C=O) charge transfer for cocrystals (I) and (II). The second order perturbation energies E^(2)^ values are indicated.

In cocrystal I, the analysis reveals electron donation from a lone pair (LP) orbital on the oxygen atoms to the antibonding π* orbitals of two C=O groups, with an associated stabilization energy of 0.22 kcal/mol, indicating weak orbital contributions to the interaction. Notably, no electron transfer to the π* orbital of the C=N bond in the oxime group is observed, consistent with the geometric features of the interaction (see Figure 8). In cocrystal II, the NBO analysis indicates electron donation from the LP orbital of the oxygen atom to the π* orbital of the nearest C=O bond, resulting in a stabilization energy of 0.30 kcal/mol, greater than that observed for cocrystal I with two *n* → π* contacts. Thus, while the NCIplot analysis confirms the LP···π nature of these interactions based on the shape of the RDG isosurface, the NBO results provide further orbital-level evidence that the *n* → π* interaction predominantly involves one of the C=O bonds in cocrystal II.

## 4 Conclusions

In this study, three new cocrystals of Oxyma-B with structurally diverse nitrogen-containing coformers, 6-methylquinoline (**I**), 2,3,5,6-tetramethylpyrazine (**II**), and 1,10-phenanthroline (**III**), were synthesized and characterized using single-crystal X-ray diffraction. All three systems exhibited rich supramolecular frameworks sustained by a combination of classical hydrogen bonds, π-stacking, CH···π, and notably, lone pair···π (LP···π) interactions. Hirshfeld surface analysis quantitatively confirmed the key contributions of these noncovalent interactions to the crystal packing.

Complementary DFT calculations offered further insight into the nature and strength of the observed interactions. Molecular electrostatic potential (MEP) surfaces helped rationalize the directionality and preference of hydrogen bonding and LP···π (or *n* → π*) interactions. QTAIM and NCIPlot analyses validated the topological features of these interactions and enabled energetic quantification of hydrogen-bonded and π-stacked cocrystallized molecular pairs. Importantly, the theoretical results revealed that the LP···π interactions in cocrystals **I** and **II** are relatively insensitive to the presence of cooperative hydrogen bonding or π-stacking, likely due to compensatory changes in electrostatic potential. In contrast, cocrystal **III** showed favorable cooperativity between hydrogen bonding and π-stacking, resulting in enhanced interaction energies.

Together, the experimental and computational results underscore the central role of Oxyma-B as a versatile building block for supramolecular design, particularly through its capacity to engage in diverse and cooperative noncovalent interactions. This work broadens the understanding of *n* → π* contacts and provides a rational framework for designing functional multicomponent crystalline materials based on Oxyma derivatives.

## Supplementary Material



## Notes

Materials, ORTEP and crystal packing representations, Fingerprint plots from Hirshfeld surface analysis. CCDC 2464146, 2464147, 2464172 contain the supplementary crystallographic data for this paper. These data can be obtained free of charge via www.ccdc.cam.ac.uk/data_request/cif, or by emailing data_request@ccdc.cam.ac.uk, or by contacting The Cambridge Crystallographic Data Centre, 12 Union Road, Cambridge CB2 1EZ, UK; fax: +44 1223 336033 (PDF)
