# Supplementary material for: Crystallographic and Computational Analysis of Oxyma B Cocrystals with Nitrogen-Containing Coformers: The Relevant Role of n → π* Interactions in Their Diverse Supramolecular Architectures
Source: Cryst Growth Des. 2025 Sep 25;25(20):8503–15. doi: 10.1021/acs.cgd.5c00884 (PMC12532365; doi:10.1021/acs.cgd.5c00884)
Supplement: Supplementary file 1 [file cg5c00884_si_001.pdf]

Crystallographic and Computational Analysis of Oxyma B cocrystals with nitrogen-containing coformers: the relevant role of n→π* interactions in their diverse Supramolecular Architectures

Mahdi Jemai,[a,b] Rafael Barbas,[c] Miquel Barceló-Oliver,[d] Houda Marouani,[b] Fernando Albericio,[e] Antonio Frontera*[d] and Rafel Prohens*[a]

a. Laboratory of Organic Chemistry, Faculty of Pharmacy and Food Sciences, University of Barcelona, Avda. Joan XXIII, 08028 Barcelona, Spain.

b. Laboratory of Material Chemistry, LR13ES08, Faculty of Sciences of Bizerte, University of Carthage, 7021 Bizerte, Tunisia.

c. Unitat de Polimorfisme i Calorimetria, Centres Científics i Tecnològics, Universitat de Barcelona, Baldiri Reixac 10, 08028 Barcelona, Spain

d. Department of Chemistry, University of Balearic Islands, 07122 Palma, Spain.

e. CIBER-BBN, Networking Centre on Bioengineering, Biomaterials and Nanomedicine, and Department of Organic Chemistry, University of Barcelona, Martí i Franqués 1-11, 08028 Barcelona, Spain.

[*] Corresponding authors at: Universitat de Barcelona. E-mail address: rafel_prohens@ub.edu (R. Prohens) and Universitat de les Illes Balears, E-mail adress: toni.frontera@uib.es (A. Frontera)

Materials

Oxyma-B was obtained from Luxembourg Bio Technologies. 6-methylquinoline (98%), 1,10-phenanthroline (99%), and 2,3,5,6-tetramethylpyrazine(98%) were used as received from Sigma-Aldrich. Ethanol (96% Technical grade) was purchased from Panreac, acetone (≥99.5%, GPR Rectapur) was purchased from VWR Chemicals, tetrahydrofuran (>99.5%, GC grade) was purchased from TCI (Tokyo chemical industry).

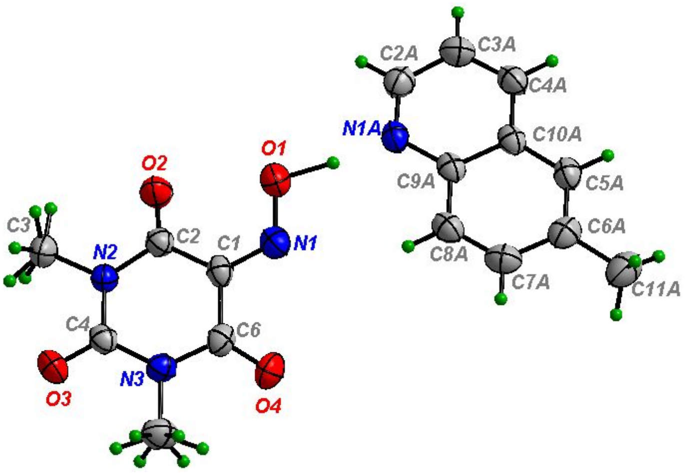

Figure.S1: ORTEP representation of 6-methylquinoline/ Oxyma-B cocrystal asymmetric unit with the labeling scheme

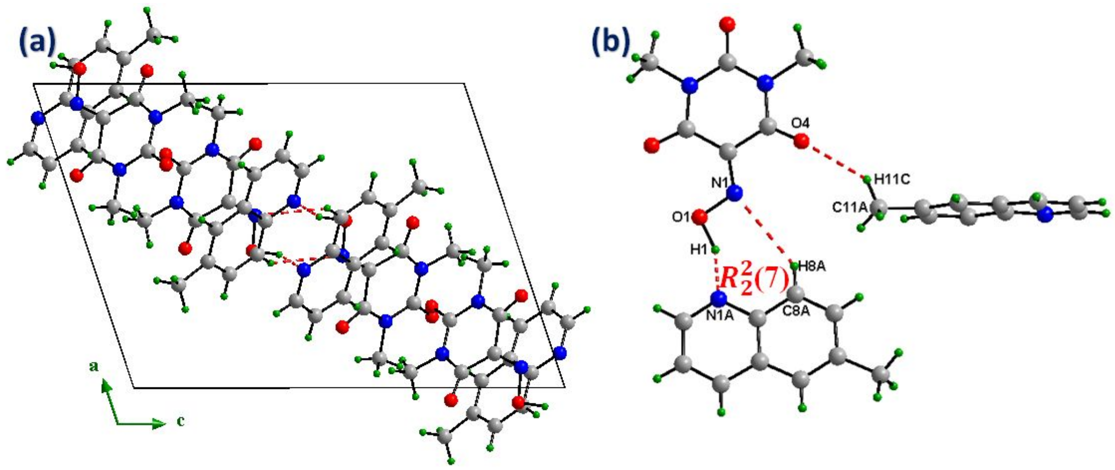

Figure. S2 (a) Crystal packing of the 6-methylquinoline/Oxyma-B cocrystal (I) along the $(\vec{a},\vec{c})$ plane, (b) Detail of the supramolecular attachment formed via OH···N and CH···O hydrogen bonding.

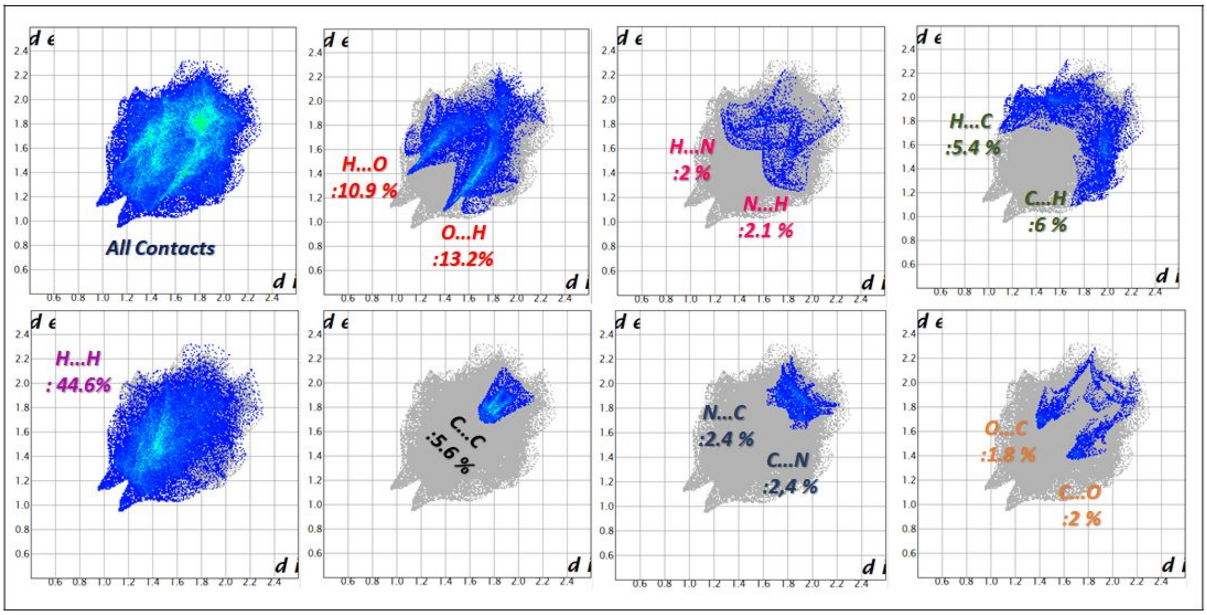

Figure. S3: Computed fingerprint plots of Co-crystal I.

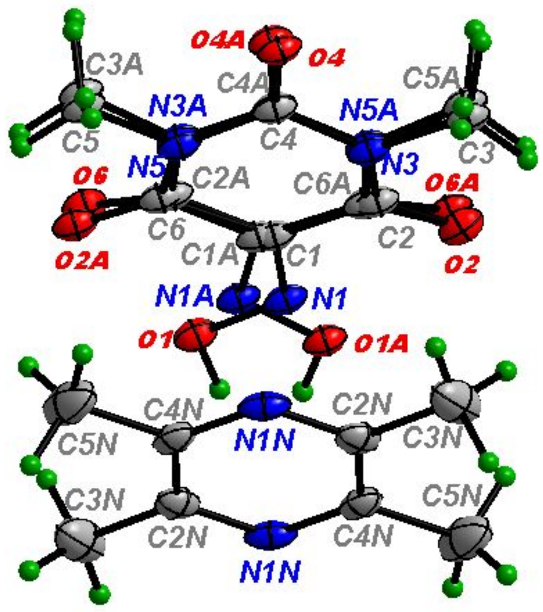

Figure S4. Ortep representation of 2,3,5,6-tetramethylpyrazine/Oxyma-B cocrystal II, where the Oxyma-B molecule exhibits positional disorder, modeled on two conformations.

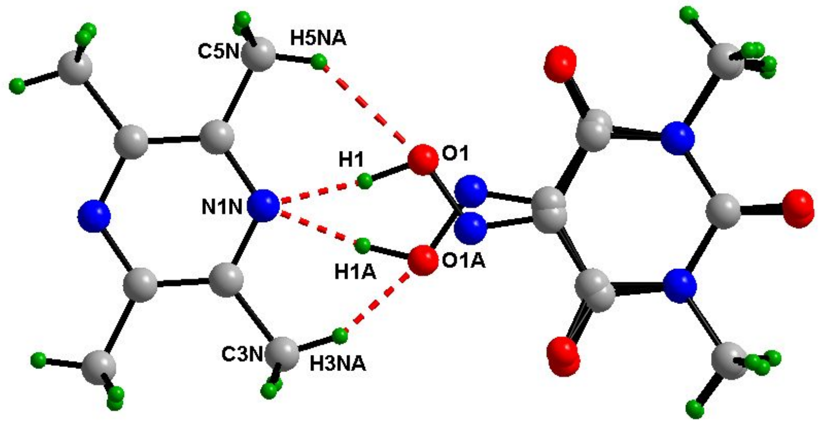

Figure S5. OH···N and CH···O hydrogen bonds involving both major and minor Oxyma-B disorder components in the cocrystal II.

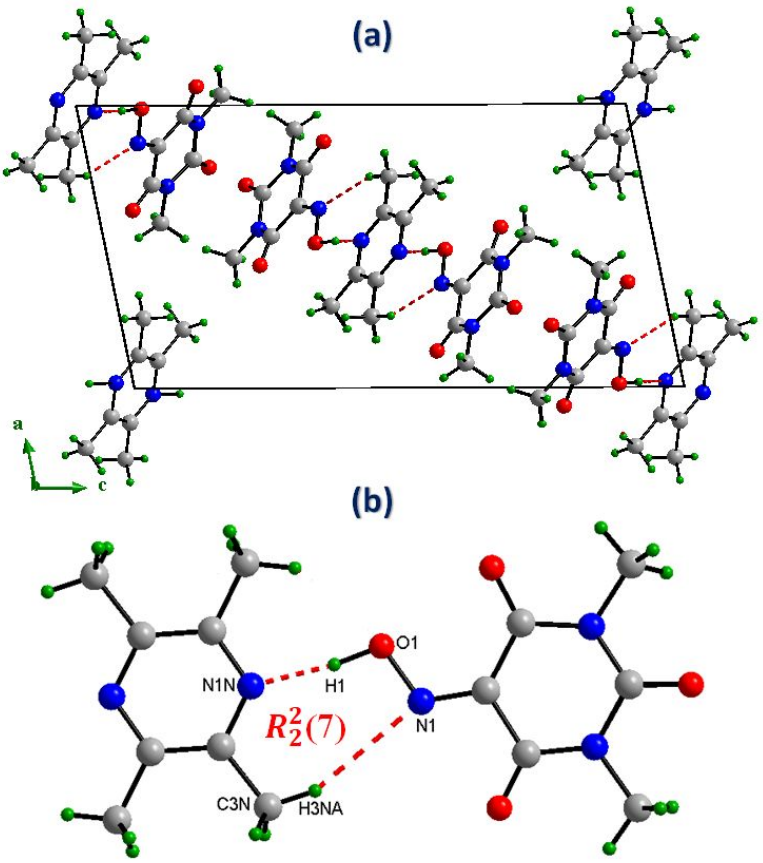

Figure S6. (a) Packing view along the $(\vec{a},\vec{c})$ plane of 2,3,5,6-tetramethylpyrazine/Oxyma-B cocrystal II, (b) Close-up view of $R_2^2(7)$ hydrogen-bonded synthons.

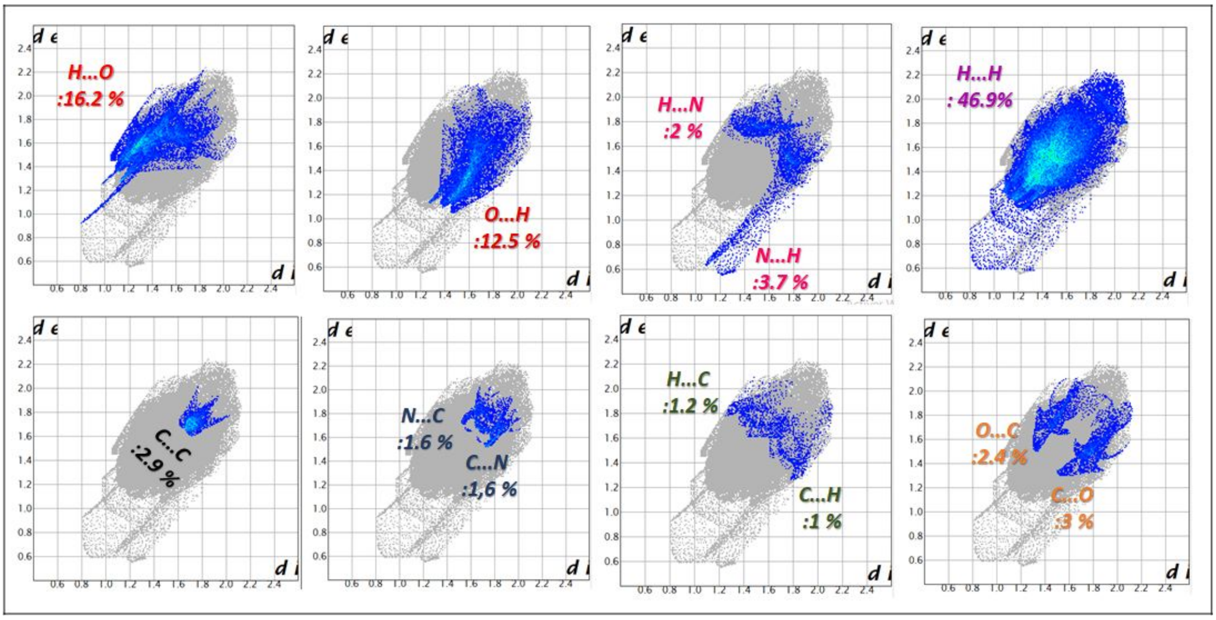

Figure. S7: Computed fingerprint plots of Co-crystal II

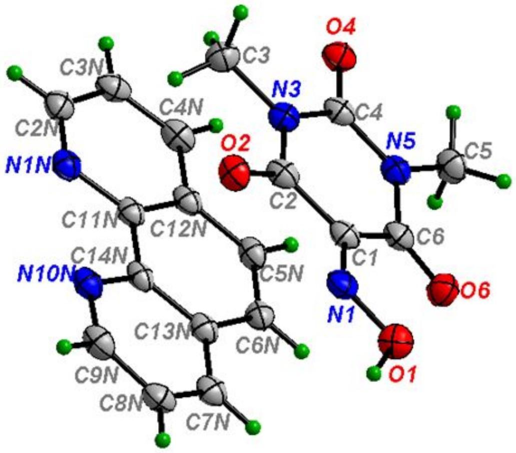

Figure S8. Asymmetric unit of 1,10-phenanthroline/Oxyma-B cocrystal (III) with atom labeling.

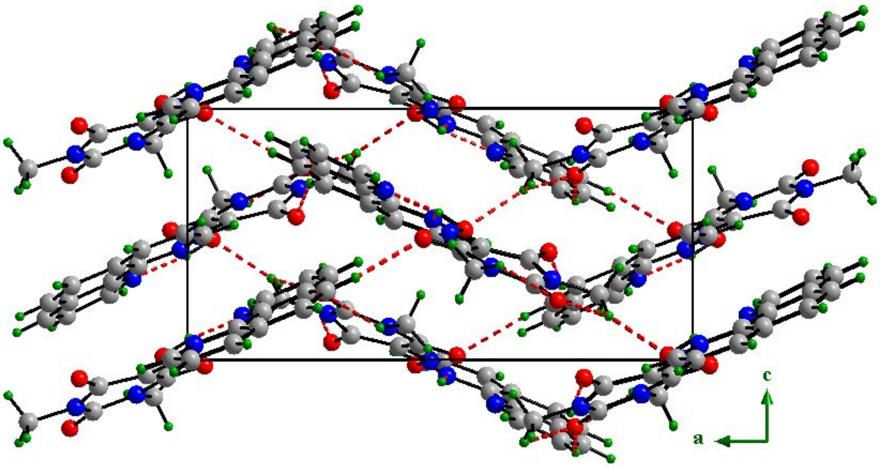

Figure S9. Projection of 1,10-phenanthroline/Oxyma-B cocrystal (III) along the $(\vec{a},\vec{c})$ plane, showing molecular chains arranged in a zigzag pattern.

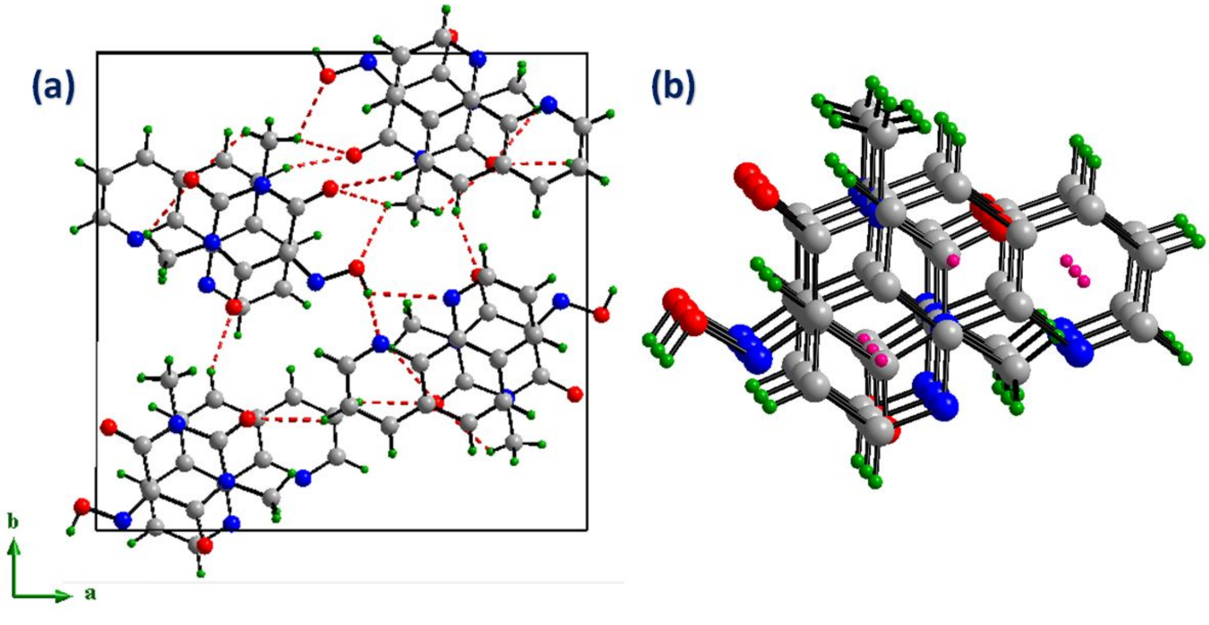

Figure S10. (a) Projection of the cocrystal III along the $(\vec{a},\vec{b})$ plane, (b) Face-to-face molecular arrangement between cocrystal III components.

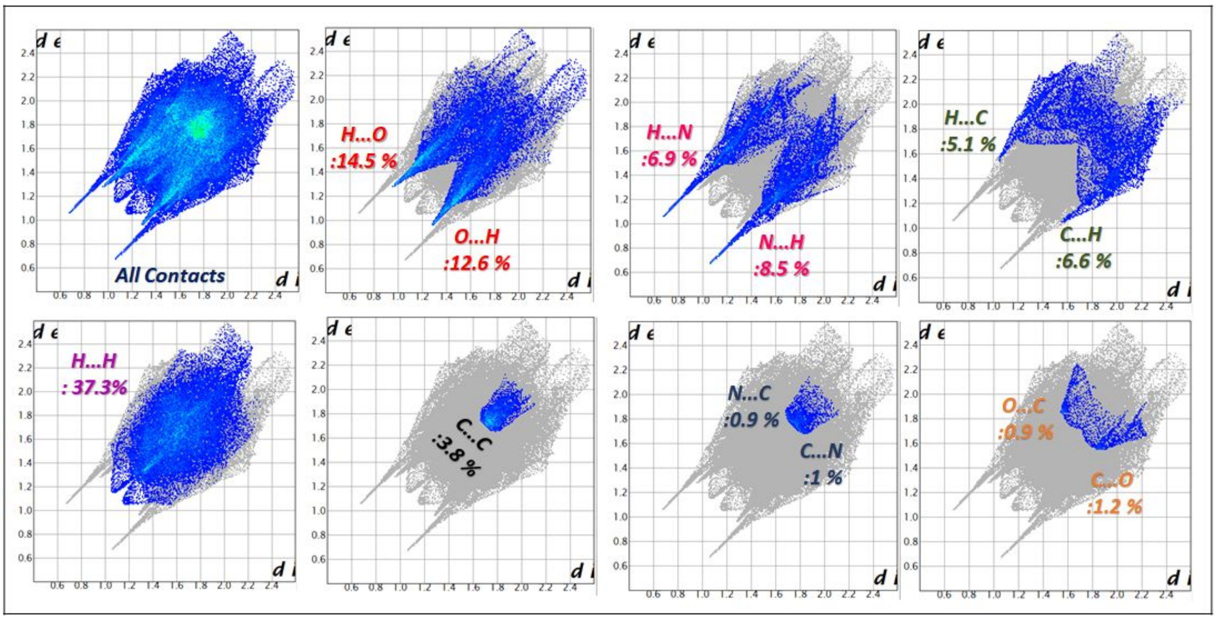

Figure S11. Computed fingerprint plots of Co-crystal III
